# Advances in RIPK1 kinase inhibitors

**DOI:** 10.3389/fphar.2022.976435

**Published:** 2022-09-28

**Authors:** Lu Chen, Xiaoqin Zhang, Yaqing Ou, Maoyu Liu, Dongke Yu, Zhiheng Song, Lihong Niu, Lijuan Zhang, Jianyou Shi

**Affiliations:** ^1^ Department of Pharmacy, Sichuan Academy of Medical Sciences & Sichuan Provincial People’s Hospital, School of Medicine, University of Electronic Science and Technology of China, Chengdu, China; ^2^ Personalized Drug Therapy Key Laboratory of Sichuan Province, School of Medicine, University of Electronic Science and Technology of China, Chengdu, China; ^3^ Department of Critical Care Medicine, Sichuan Academy of Medical Sciences & Sichuan Provincial People’s Hospital, Affiliated Hospital of University of Electronic Science and Technology of China, Chengdu, Sichuan, China; ^4^ Department of Pharmacy, The Affiliated Chengdu 363 Hospital of Southwest Medical University, Chengdu, Sichuan, China; ^5^ Suzhou University of Science and Technology, Suzhou, Jiangsu, China; ^6^ Institute of Laboratory Animal Sciences, Academy of Medical Sciences and Sichuan Provincial People’s Hospital, Chengdu, Sichuan, China

**Keywords:** inhibitor, receptor interacting protein 1 (RIP1), programmed necrosis, necrosis, RIP1 (RIPK1)

## Abstract

Programmed necrosis is a new modulated cell death mode with necrotizing morphological characteristics. Receptor interacting protein 1 (RIPK**1**) is a critical mediator of the programmed necrosis pathway that is involved in stroke, myocardial infarction, fatal systemic inflammatory response syndrome, Alzheimer’s disease, and malignancy. At present, the reported inhibitors are divided into four categories. The first category is the type I ATP-competitive kinase inhibitors that targets the area occupied by the ATP adenylate ring; The second category is type Ⅱ ATP competitive kinase inhibitors targeting the DLG-out conformation of RIPK**1**; The third category is type Ⅲ kinase inhibitors that compete for binding to allosteric sites near ATP pockets; The last category is others. This paper reviews the structure, biological function, and recent research progress of receptor interaction protein-1 kinase inhibitors.

## 1 Introduction

Unlike apoptosis, cell necrosis is an unregulated form of cell death brought about by external physical and chemical stress (Linkermann et al., 2012; Weinlich et al., 2016). However, in recent years, studies have found some distinct signal transduction pathways that are programmed to regulate cell necrosis, such as programmed necrosis, pyrolysis, and iron death. Programmed necrosis is a new type of cell death that can be regulated and characterized by necrotizing morphology (Maria, 2011; Morrice et al., 2017). In the regulation of programmed necrosis, a class of proteins occupies an important position in the signaling pathway, namely receptor-interacting protein 1 (RIPK1) (Christofferson et al., 2012; Vasilikos et al., 2017). RIPK1 is an intracellular adaptor that regulates inflammation, apoptosis, and necrosis processes by transmitting signals from receptors, and exerts kinase-independent protective functions under specific conditions (Ofengeim and Yuan, 2013; Chen et al., 2019; Ueta et al., 2019). Existing articles have reviewed the structure of previous RIPK1 inhibitors (Fang et al., 2021), kinase binding patterns (Martens et al., 2020) and the relationship between RIPK1 and disease (Zhuang and Chen, 2020). In this paper, the molecular structure and function of RIP1, the relationship between RIP1 and disease, the existing RIP1 kinase inhibitors and their structure and structure-activity relationship were summarized and analyzed, and some views on the future development of these RIP1 kinase inhibitors were put forward.

## 2 Molecular structure and function of RIPK1

### 2.1 Molecular structure of RIPK1

**FIGURE 1:**
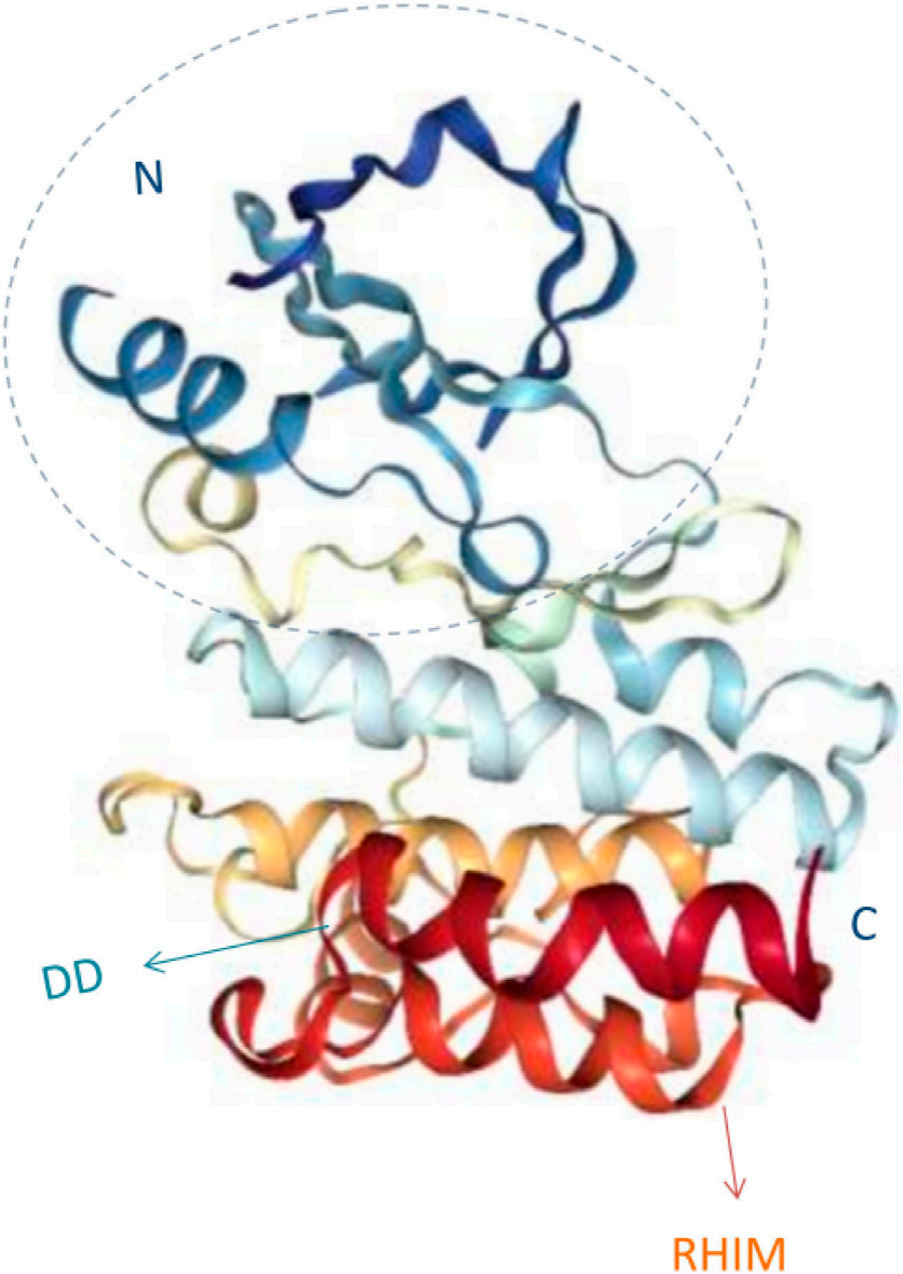
3D structure diagram of RIPK**1**.

RIP is a serine/threonine protein kinase. It was known that there were seven members of the RIP family, among which RIPK**1** and RIPK**3** are the most widely studied in necrosis (Berghe et al., 2014; Boris, 2016). They have a homologous N-terminal kinase domain but different recruitment domains in their structure (Wim, 2009) (Figure 1). The molecular structure of RIPK**1** consists of N-terminal kinase domain, an intermediate domain, and a carboxyl-terminal death domain (Lei et al., 2018). The N-terminal kinase domain can catalyze the autophosphorylation of RIPK**1** of the serine/threonine residue site, which has N-lobe, a C-lobe, and an intervening activation loop (also known as the T-loop) (Xie et al., 2013). Similar to other protein kinases, the N-lobe comprises an antiparallel, five-stranded β sheet and an activation helix (commonly known as the alpha C-helix); the C-lobe contains six α-helices and a pair of β strands; the ATP hydrolysis binding region is highly conserved and consists of a catalytic triplet composed of Lys45-Glu63-ASP156, a P-loop composed of amino acid residues at positions 24–31, and a catalytic loop region composed of amino acid residues at positions 136–143 (McQuade et al., 2013). The C-terminal death domain interacts with tumor necrosis factor (TNF)-related apoptosis-inducing ligand receptor (TNF related apoptosis inducing ligand receptor, TRAILR)-**1**, TNF receptor (TNFR)-**1** and TRAILR**2**, as well as with Fas-related death domain (FADD), TNF receptor-related death domain (TRADD) and other molecules containing death domain. The RIP homotypic interaction motif (RHIM) is a segment of the intermediate domain. RHIM, composed of about 35 amino acids, mediates homologous interaction between RIPK**1** and RIPK**3** to activate the downstream signaling pathway (Lukens et al., 2013; Pasparakis and Vandenabeele, 2015).

### 2.2 Biological function of RIPK1

**FIGURE 2:**
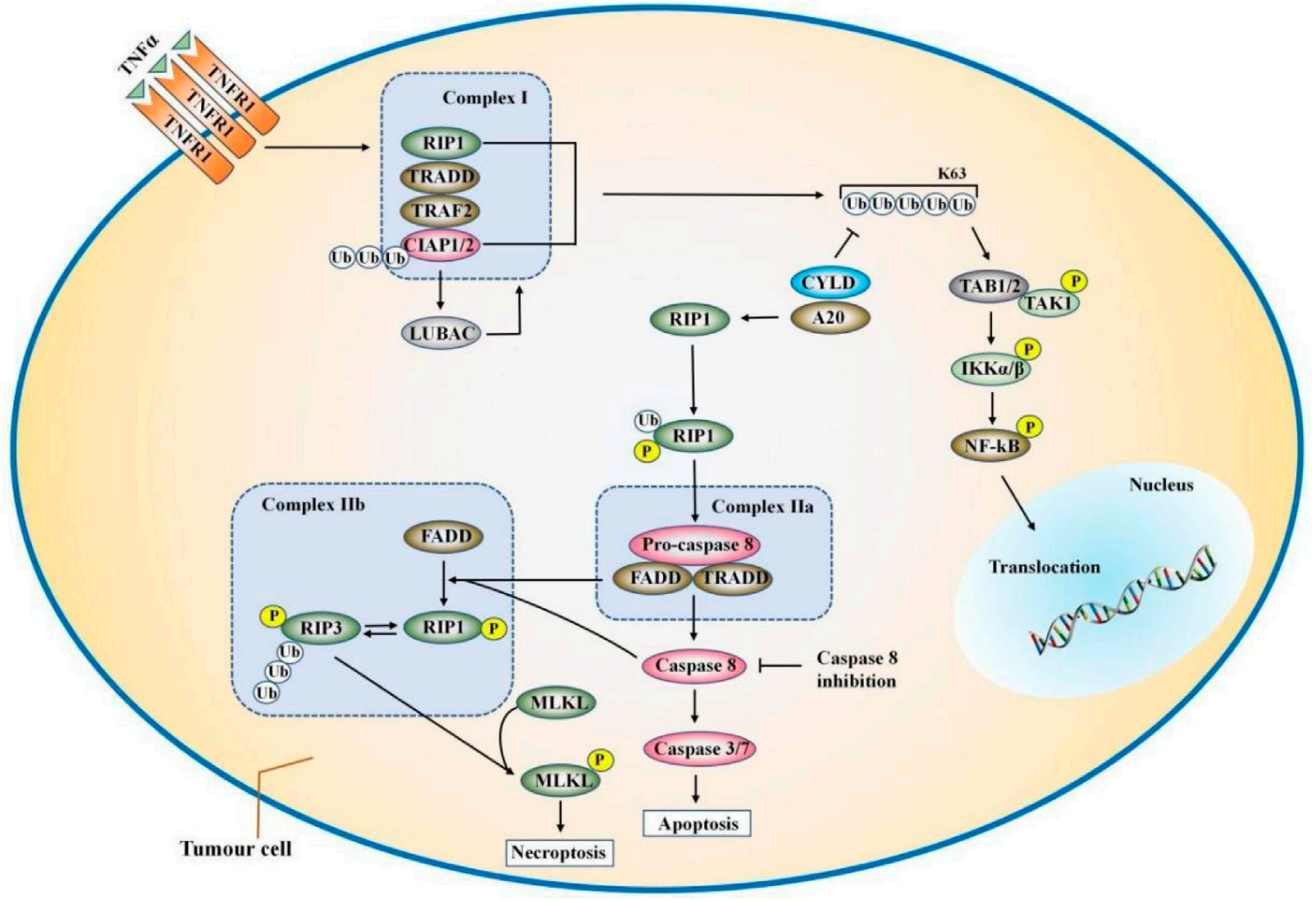
The combination of TNFα and TNFR**1** can trigger a variety of signaling pathways, including NF-κB, apoptosis and necrosis. TNFα induces the formation of complex I, which is composed of RIPK**1**, TRADD, TRAF**2**/**5**, LUBAC, and cIAP**1**/**2**. In complex I, cIAP**1**/**2**, and LUBAC induce ubiquitination of RIPK**1**. The ubiquitination of the Lys**63** domain of RIPK**1** further promotes the formation of IKK and TAK complexes, which ultimately lead to the activation of the NF-κB pathway and cell survival. CYLD or A**20** deubiquitinates RIPK**1** and induced the separation of TRADD and RIPK**1** from TNFR1, thereby forming complex IIa or complex IIb. FADD and pro-caspase-8 are called into TRADD and RIPK**1** to form complex IIa, which activates caspase-8 through oligomerization and cleavage. When the activity of CIAPS, TAK**1**, NEMO is inhibited or the expression is knocked down, complex **II**b is formed and caspase-**8** is activated. Complex IIb contains TRADD, RIPK**1**, FADD, and pro-caspase-**8**. Then, caspase-**8** induces apoptosis. When the activity of caspase-**8** is blocked, such as cFLIP or the pan-caspase inhibitor zVAD-fmk, the cell will go to the necrotic pathway.

TNF- α and TNFR1 first form a trimer, and then recruit proteins containing the death domain (DD) to form complex Ⅰ (Hsu et al., 1995; Micheau and Tschopp, 2003; Rangamani and Sirovich, 2010) (Figure 2). These proteins are composed of TNFR1-associated death domain protein (TRADD), RIPK**1**, and the E**3** ubiquitin ligases TNF-receptor-associated factor **2** (TRAF**2**), the cellular inhibitors of apoptosis (cIAP**1** or cIAP**2**), and the linear ubiquitin chain assembly complex (LUBAC) (Pasparakis and Vandenabeele, 2015). C-IAP**1**/**2** promote ubiquitination of themselves and RIPK**1** through K**63**, K**48**, and K**11** chains (Witt and Vucic, 2017). Polyubiquitin chains conjugated by c-IAP**1**/**2** allow the recruitment of linear ubiquitin assembly complex (LUBAC) (Gerlach et al., 2011). LUBAC generates exclusively linear ubiquitin chains on several molecules including RIPK**1**, TNFR1, TRADD, and NEMO (Tokunaga and Iwai, 2012; Witt and Vucic, 2017). Ubiquitination of the Lys**63** domain of RIPK**1** promotes the recruitment of Ikappa B kinase (Ikappa B kinase, IKK) and transforming growth factor kinase (TGF β-activated kinase, TAK) into IKK complexes (IKK α and IKK β) and β activated kinase (TAK) complexes [TAK**1** and TAK**1** binding protein (TAB) one and 2], thereby activating the nuclear factor-kappa B (NF-κB) pathway (Xiao et al., 2015; Chen et al., 2019). In complex **I**, IKKα/IKKβ can also directly phosphorylate RIPK**1** and inactivate it, resulting in a decrease in the ability of RIPK**1** to bind to FADD/caspase-**8** and induce apoptosis (Dondelinger et al., 2015). Linear ubiquitination of RIPK**1** and other TNFR1-related proteins further enhances TNF-stimulated NF-κB and mitogen-activated protein kinases (MAPK) signaling (Christofferson and Yuan, 2010; Zarrin et al., 2021). Complex I mediates NF-κB and MAPK signaling, contributing to cell survival or other non-death functions (Chen et al., 2016; Liu et al., 2019).

Deubiquitin enzymes A**20** and Cylindromatosis (CYLD) can decompose the polyubiquitin chains of RIPK**1** and other ubiquitin components of complex **I** (Kelly, 2012; Moquin et al., 2013; Emmerich et al., 2016), leading to the formation of complex II to prompt cell apoptosis and necroptosis. According to the different protein composition, complex II has two different forms, namely complex Ⅱa and complex Ⅱb. After dissociating from TNFR1, TRAdd recruits Fas-associated protein with death domain (FADD) and further promotes the recruitment and activation of caspase-**8** to form the complex IIa (Wang L. et al., 2018). Complex Ⅱa contains FADD, TRAdd, and pro-caspase-**8**. When the activity of CIAPS, TAK**1**, NF-κB essential modulator (NEMO) is inhibited or the expression is knocked down, RIPK**1** is released from complex **I** by TNF induction and forms complex **II**b with RIPK**3**, FADD, and pro-caspase-**8** to promote RIPK**1** and caspase-**8** dependent apoptosis (Benetatos et al., 2014). First, caspase-**8** is recruited into FADD in the form of pro-caspase-**8** and more pro-caspase-**8** molecules are recruited to form homodimer (Newton et al., 2014). Then two pro-caspase-**8** proteins are cleavaged with aspartic acid, leading to the maturation of pro-caspase-**8** into caspase-**8** (Newton et al., 2014). Subsequently, caspase-8 activate caspase-**3**/**7** leading to apoptosis (Newton et al., 2014). Although RIPK**1** participates in the apoptosis pathway, it is not an essential factor in the process of apoptosis.

However, when caspase-**8** is inhibited, such as by the caspase-**8** inhibitor z-VAD, the cells will go to the necrotic pathway. RIPK**1** is activated by autophosphorylation, which leads to the recruitment and phosphorylation of RIPK**3** and mixed lineage kinase domain-like protein (MLKL), and finally forms a complex called “necrosome” (Newton et al., 2014; Shen et al., 2017). The autophosphorylation of RIPK**1** is the key to the recruitment of RIPK**3**. RIPK**1** is phosphorylated by RIPK**3** after they are bound by RHIM. Finally, the autophosphorylation and dephosphorylation of RIPK**1**/RIPK**3** promote the recruitment of MLKL (Newton, 2015). The kinase activity of RIPK**1** is required for the interaction of RIPK**1** with RIPK**3**, which may be because the conformational changes caused by RIPK**1** autophosphorylation expose the interaction between RHIM and DD at the c-terminal of RIPK**1** (McQuade et al., 2013; Newton, 2015). After being phosphorylated, MLKL is oligomerized and transferred to the cell membrane, permeabilizing the cell membrane, destroying the integrity of the membrane, and ultimately leading to programmed cell death (Grootjans et al., 2017). It can be seen that RIPK1 simultaneously transmits signals that promote cell survival, cell apoptosis, and programmed cell death (Saddoughi et al., 2013). These different cellular functions are mediated by differentially modifie. These different cellular functions are mediated by different modifications of RIPK**1**.

## 3 RIPK1 and diseases

RIPK**1** plays an important role in the pathogenesis and evolution of many diseases, so it is a major target for drug development at present. RIPK1 was overexpressed in tissues of patients with ischemic stroke, atherosclerosis, and aortic aneurysm (Linkermann et al., 2012; Dannappel et al., 2014). And in mouse models, inhibition of RIPK**1** reduced the severity of the disease (Takahashi et al., 2014; Wang et al., 2017). It has been found that many diseases are related to RIPK**1**/programmed necrotic pathway (Cao and Mu, 2021).

### 3.1 RIP1 with inflammatory disease

Genetic loss of RIPK**1** kinase activity relieved the symptoms of hypothermia. RIPK**1** kinase activity completely saved the inflammation of cpdm mice, indicating that RIPK**1** kinase had a pro-inflammatory effect in addition to driver-induced necrosis (Berger et al., 2014). The study highlighted the clinical relevance of RIPK**1** kinase inhibition in sepsis and revealed the possibility of targeting RIPK**1** in the programmed necrosis pathway as a potential therapeutic target for SIRS and sepsis (Gupta et al., 2018). Inhibition of RIP1 by Nec-**1** reduced the expression of reactive oxygen species (ROS) and Nicotinamide Adenine Dinucleotide Phosphate Oxidase 4 (NoX-4), improved hbX-induced oxidative stress, inhibited the production of pro-inflammatory cytokines interleukin-6 (IL-6), interleukin-6 (IL-8), CXCL2 and secretion of high Mobility Group Protein B1 (HMGB1) (Xie and Huang, 2019). These results suggested that RIP1 was involved in hbX-induced cytotoxicity and inflammatory responses in normal human liver cells. GNE684 could effectively block Sharpin mutant mice (Cpdm; Chronic proliferative dermatitis) showed skin inflammation and liver immune cell infiltration, suggesting that inhibition of RIP1 can treat skin inflammatory diseases. However, in wild-type, RIP1 KD, and RIP3 KO mice, vaccinia virus or murine herpes virus MHV68 infection also resulted in similar viral clearance, suggesting that RIPK1 was not associated with certain viral infections (Webster et al., 2020). Nec-**1** significantly decreased the levels of interleukin 1β, IL-6 and TNF-α in RIPK**1**, RIPK**3**, MLKL, phosphorylated (P) -RIPK**3**, and P-MLKL as well as periventricular regions, but did not reduce ependymal cilium damage or brain water content. These results suggested that Nec-**1** could prevent local inflammation and microglial activation induced by IVH, but could not greatly improve the prognosis (Liu et al., 2022). Nec-**1** inhibited RIPK**1** activity, caspase-1 cleavage induced by er stress and IL-1β secretion in bone marrow derived macrophages (BMDMs) and J774A were significantly reduced. Mitochondrial fission factor dynamic related protein 1 (DRP1) and ROS may be downstream effeffers of RIPK**1**, mediating the activation of inflammasosomes (Tao et al., 2018). This result demonstrated that RIPK**1** played a key role in er stress-induced inflammatory responses and served as a potential drug target for the treatment of er stress-induced inflammatory diseases.

### 3.2 RIP1 with ischemic injury

Necrotic apoptosis mediated by RIPK**1**/RIPK**3**/MLKL signaling is a key process in the development of acute ischemic stroke. Insoluble RIPK**1**, RIPK**3** and MLKL were detected in the infarct area of acute ischemic stroke mice, suggesting that necrotic apoptosis is associated with ischemic stroke. In RIP1 kinase death mutant mice, middle cerebral artery occlusion/reperfusion (MCAO/R) reduced infarct size and improved neurological function, and the mice also showed less inflammatory response in the infarct area, suggesting that necrotizing apoptosis and its concomitant inflammatory response can lead to acute injury after ischemic stroke (Zhang et al., 2020). RIPK**1** knockdown or necrostin-1 therapy reduced infarct volume, improved neurological deficits, increased levels of microtubule-associated protein 2 (MAP2) and glial fibrillary acidic protein (GFAP), and attenuated neuronal or astrocyte ischemic cortical necrotic cell death (Ni et al., 2018). RIPK**1** participateed in astrocyte hyperplasia and formation of glial scar by damaging the normal response of astrocytes and enhancing the VEGF-D/VEGFR-3 signaling pathway of astrocytes. Therefore, inhibition of RIPK**1** partially promoted the recovery of brain function by inhibiting astrocyte hyperplasia and formation of glial scar (Zhu et al., 2021). RIPK**1** KD rats were protected from a range of behavioral, imaging, and histopathological endpoints in a rat model with RIPK**1** (D138N) mutant knock-in, and the accumulation of inflammatory and neuronal damage biomarkers was reduced in RIP1 KD rats (Stark et al., 2021). Inhibition of RIPK**1** inhibited programmed necrosis after myocardial ischemia-reperfusion, reduced myocardial infarction size, reduced inflammatory response, reduced the production of ROS, and then improved the function of cardiac remodeling in the infarcted area (Oerlemans et al., 2012). In the rat model of intestinal ischemia/reperfusion (I/R) injury, the expressions of RIP**1/3** and p-MLKL were significantly increased in tissues, suggesting necrosis and acute distal liver function damage (Wen et al., 2020). Inhibition of the necrosis pathway ameliorated acute liver injury caused by hepatocyte necrosis. In mice with retinal degeneration RD1 and acute retinal nerve injury, necrotic microglia released various pro-inflammatory cytokines and chemokines to contribute to retinal inflammation. Blocking necrotic apoptosis with Nec-**1** inhibited microglia-mediated inflammation (Huang et al., 2018).

### 3.3 RIP1 with neurodegenerative disease

In the cortex and hippocampus of APP/PS1 double transgenic mice, RIPK**1** inhibitors dropped out the levels of β-amyloid plaque, oligomer, and hyperphosphorylated tau protein, but did not affect the production of β-amyloid protein and changed the level of apoptosis marker protein (Strilic et al., 2016; Yang et al., 2017). RIPK**1** was highly expressed in microglia in Alzheimer’s disease (AD). Inhibition of RIPK1 by amyloid precursor protein (APP)/premature aging protein 1 (PS1) transgenic mouse models reduced amyloid burden, inflammatory cytokine levels, and memory deficits, and also promoted microglial degradation of Aβ *in vitro* (Ofengeim et al., 2017). These data suggested that RIPK**1** mediated a key checkpoint during the transition to disease-associated microglia (DAM) state (Ofengeim et al., 2017). Sod1-tgs mice with genetically inactivated RIPK**1** kinase activity did not improve muscle strength or nerve function, motor neuron degeneration or neuroinflammation, and there was no accumulation of phosphorylated RIPK**1** in the spinal cord of ALS patients. In a toxic model of dopaminergic neurodegeneration, genetic inactivation of RIPK**1** kinase activity ameliorates loss of the neurotransmitter dopamine (Dominguez et al., 2021). These results suggested that RIPK**1** kinase activity was unnecessary for the pathogenesis of SOD1-TG mice, while inhibition of kinase activity may be beneficial for acute injury models (Liang et al., 2019).

### 3.4 RIP1 with oncology

The expression of key regulators of the necrotic apoptosis pathway was generally down-regulated in cancer cells, suggesting that cancer cells may survive by avoiding necrotic apoptosis (Gong et al., 2019). However, in some types of cancer, expression levels of key mediators are elevated (Gong et al., 2019). Necrotic apoptosis could induce a strong adaptive immune response and prevent tumor progression. However, inflammatory responses to recruitment also promoted tumgenesis and cancer metastasis, and necrotizing apoptosis created an immunosuppressive tumor microenvironment (Gong et al., 2019). Studies had shown that RIPK**1** expression was significantly reduced in colon cancer tissues compared with adjacent normal colon tissues, thus impounding the cancer cell response to programmed necrosis (Moriwaki et al., 2015). Wang et al. reported that RIPK**1** inhibitors reduced tumor burden and prolonged survival in orthotopic PDA tumor cells derived from KPC mice, and also prevented tumor metastasis (Wang W. et al., 2018). But Patel et al. found that in two different pancreatic duct adenocarcinoma models (pancreatic tumor models driven by mutant Kras and B16 melanoma model), RIPK**1** inhibitors did not slow tumor growth, and RIPK**1** inactivation did not lead to macrophage reprogramming and/or STAT1 activation (Patel et al., 2020). The inconsistency between the results of the two studies is due to different RIPK**1** inhibitions selected and different animal models. The study of the relationship between RIPK**1** and disease, especially the relationship between tumor growth, requires highly specific RIPK**1** inhibitions and animal models. To date, clinical trials of RIPK1 inhibitors for the treatment of solid tumors have not succeeded, which creates doubt over the use of RIPK1 inhibitors as anticancer treatments (Martens et al., 2020). It was found that shikonin induced RIPK**1** expression in glioma cells, thereby depleting glutathione (GSH), promoting hydrogen peroxide production and inhibiting glycolysis (Lu et al., 2018). Rubraca inhibitd the proliferation of ovarian cancer SKOV3 and A2780 cells by upregulating the expression of RIPK**1** and RIPK**3** proteins to activate necrotic apoptosis (Wang et al., 2020).

Based on these studies, the development of therapeutic drugs targeting RIPK**1** is a new strategy to block cell death in the process of ischemic cardio-cerebrovascular diseases, inflammation, neurodegenerative diseases, and so on (Manguso et al., 2017). However, the role of RIPK**1** kinase in various diseases is not consistent. It is still controversial whether RIPK**1** should be used as a therapeutic target for cancer. Therefore, studying the pathological mechanism of RIPK**1**’s participation in various diseases is the focus of researchs. In addition, RIPK**1** prevented embryonic and postnatal death by cutting off two different cell death pathways, FADD/Caspase-8-mediated apoptosis and RIPK**3**-mediated necroptosis (He et al., 2009). Therefore, the role of RIPK**1** in various diseases remains to be further explored. The specific role of RIPK**1** in the occurrence and development of diseases requires a detailed study of RIPK**1**’s role in the necrosis process, that is, under what conditions RIPK**1** is involved in apoptosis, necrosis, and cell survival.

## 4 Advances in RIPK1 kinase inhibitors

### 4.1 Type Ⅰ ATP enzyme inhibitors

**FIGURE 3:**
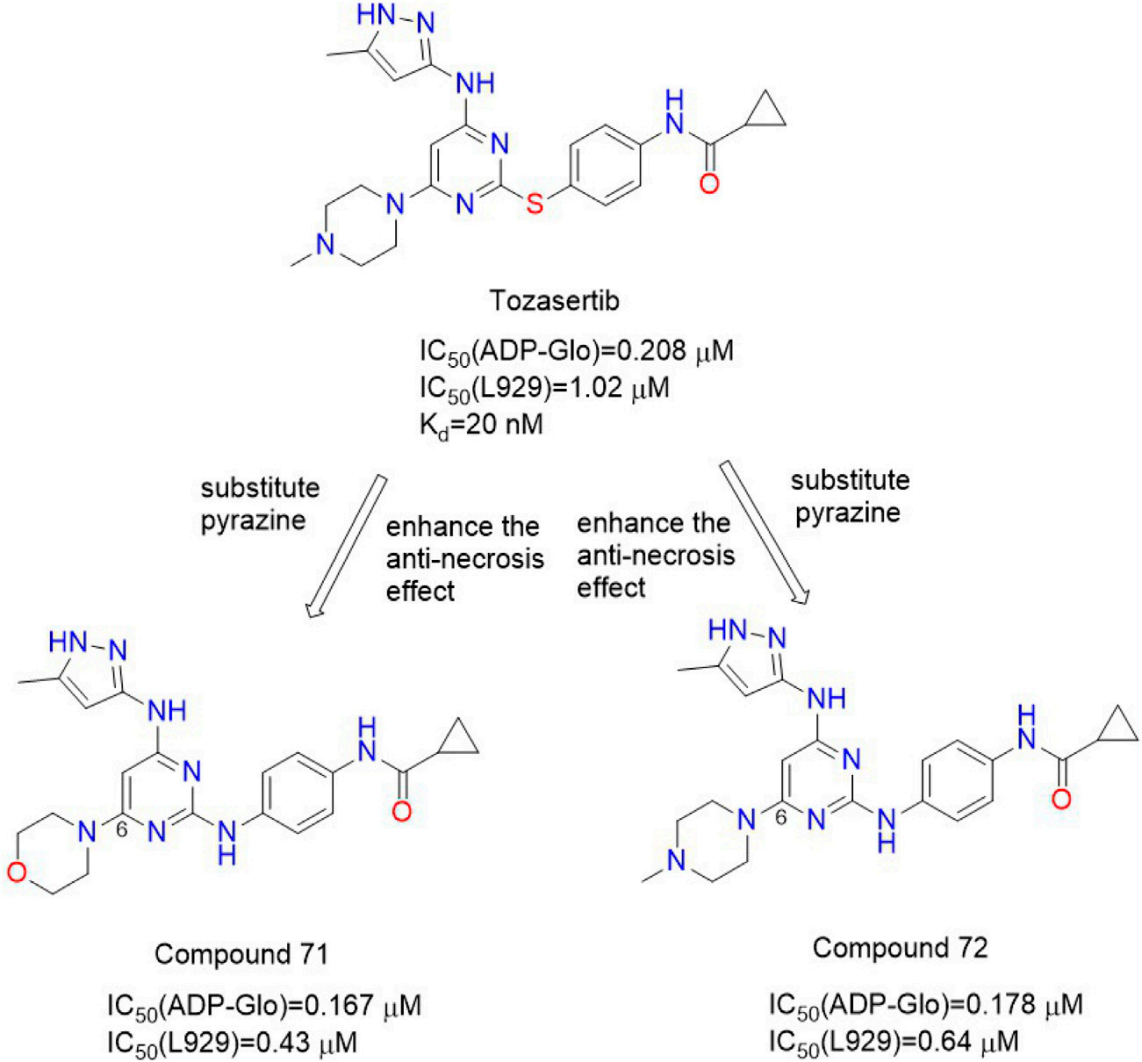
Structures of Compound **71** Compound **72** and their bioactivities.

Hofmans et al. found that Tozasertib, the type I Aurora A/B/C kinase inhibitor, had a very high affinity for RIPK**1**, with a Kd value of 20 nM (Divert, 2018). Tozasertib was originally an Aurora kinase (AurK) inhibitor. AurK was key to the process of cell division (Harrington et al., 2004). Thus, inhibition of AurK by Tozasertib led to cellular abnormalities. Compound **71** (Figure 3) and Compound **72** (Figure 3) were obtained through structural modification to modify the kinase inhibition spectrum, which could inhibit RIPK**1** in enzymatic experiments with IC_50_ (ADP-Glo) values of 0.167 μM and 0.178 μM, respectively. Compound **71** and Compound **72** had good inhibitory effects on TNF-induced necrosis, with IC_50_ (L929) values of 0.43 μM and 0.64 μM, respectively. Their inhibitory effect on AURK was significantly reduced, and they performed better than Tozasertib in the TNF-induced Systemic Inflammatory Response Syndrome (SIRS) mouse model (Divert, 2018).

Structure-activity relationship analysis showed that The linkage of cyclopropyl and cyclohexyl substituents in the amide portion had a good inhibitory effect on TNF-induced necrosis. The 4-position hydrogen and methyl substitution on the pyrazole ring were well tolerated, and hydrogen substitution was more effective than methyl substitution. It was most effective to connect N-methyl piperazine, morpholine ring, or piperidine ring at the 6-position of the pyrimidine ring. The connecting atoms between the benzene ring and pyrimidine ring had different conjugation with the aromatic ring due to different electronic properties, thus changing the rigidity of the general structure of the aromatic ring system and affecting the conformational preference of the system. Therefore, the selection of connecting atoms could reduce the cellular effects related to the inhibition of AURK. The nitrogen bond was more strongly conjugated to the aromatic ring system, which increased the rigidity of the general structure of the system, so that the nitrogen bond compound could not be cooperated with AURK in the same conformation. In the Tozasertib: AURK A: TPX**2** cocrystal structure, there was a 98 °twist angle in the crystal structure of tozasertib (Divert, 2018). The two aromatic rings connected by sulfur atoms were approximately perpendicular to each other and bound to AURK (Divert, 2018). Compared with the sulfur bond system, the most stable conformation of the nitrogen bond system was the complete planar conformation, which can not be combined with AURK.

Molecular simulation studies showed that due to conformational changes, nitrogenous compounds could not bind to AURK in the same conformation as Tozasertib. For compound **71** and compound **72**, the connecting atoms between the two six-membered aromatic rings were changed from sulfur to nitrogen. This makes them less selective for AURK. The binding mode of Tozasertib is similar to that observed in the eutectic structure of Tozasertib and AURKA, that is, the three hydrogen bonds to the hinge motif are conservative and the phenyl ring interacts with the G-loop (Divert, 2018).

### 4.2 Type II ATP enzyme inhibitors

Type II enzyme inhibitors occupy a hydrophobic pocket close to the ATP binding pocket (Alexander et al., 2015). The phenylalanine movement of the DFG motif (Asp-Phe-Gly) in the A-loop produces a unique DFG-out conformation, which is an inactive Kinase status (Zhang et al., 2016). This rearrangement creates a new hydrophobic pocket, also called an allosteric site. In RIPK**1**, the DFG (Asp-Phe-Gly) motif on A-loop is Asp-Leu-Gly (DLG). These inhibitors generally have a heterocycle that forms a hydrogen bond with the hinge residues of the kinase, a hydrophobic group that occupies a new allosteric site, and urea or amide groups in the middle that have additional hydrogen bonding interactions with the highly conserved glutamic acid side chains of the Asp backbone NH and α-C helix.

#### 4.2.1 1-Aminoisoquinolines

**FIGURE 4:**
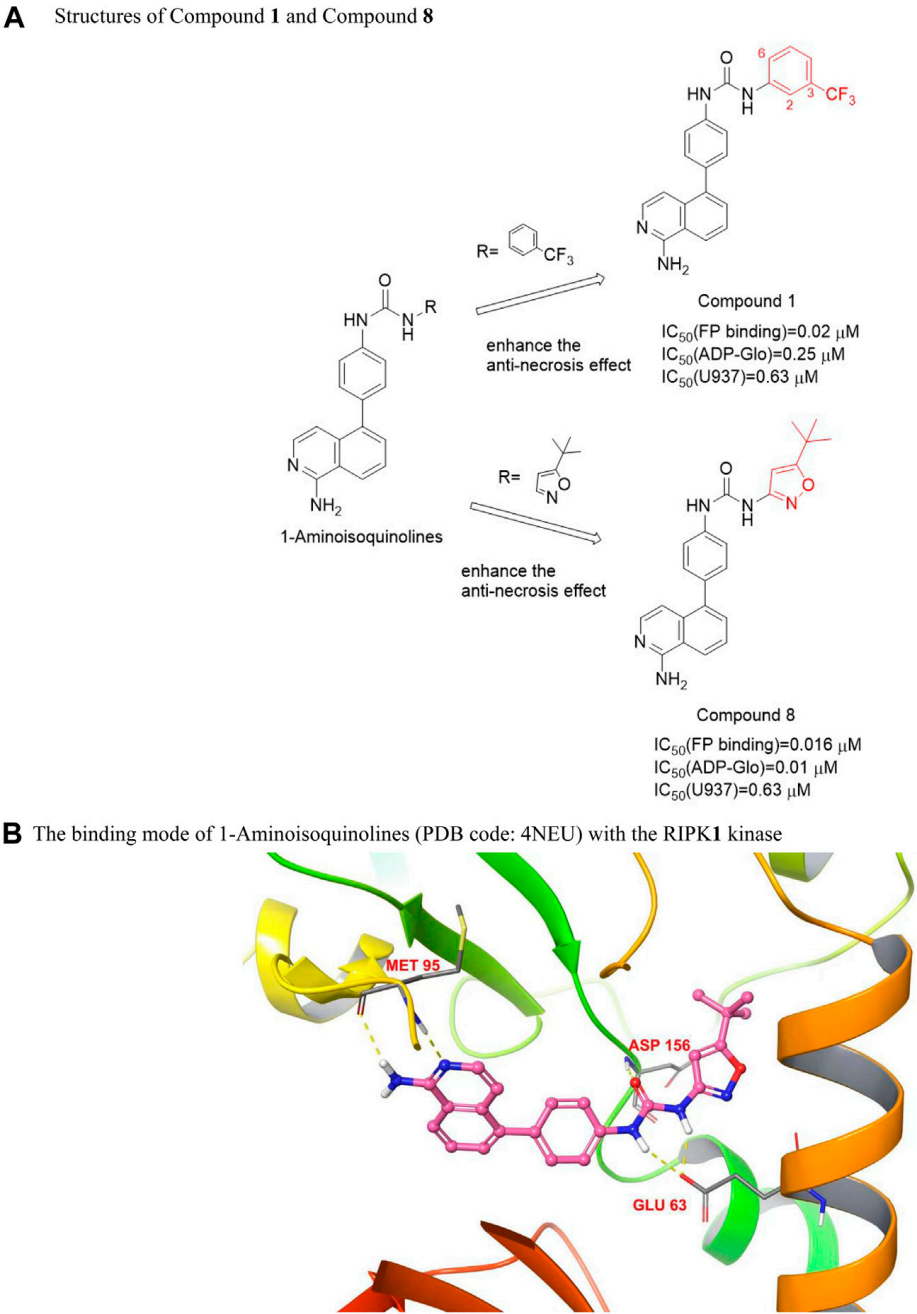
Structures of Compound **1** and Compound **8** and their bioactivities and the binding mode of 1-Aminoisoquinolines (PDB code: 4NEU) with the RIPK**1** kinase. **(A)**. Structures of Compound **1** and Compound **8**. **(B)**. The binding mode of 1-Aminoisoquinolines (PDB code: 4NEU) with the RIPK**1** kinase

These compounds had 1-aminoisoquinoline ring as hinge binder. The m-trifluoromethylphenyl substituent [IC_50_ (U937) = 0.63 μM], as a hydrophobic aryl group, targeted the conserved hydrophobic pocket on the kinase allosteric site. Except for the m-substituent of trifluoromethyl, the other substituents on the benzene ring will lead to a decrease in activity, substituting fluorine at site 2 causes most of the activity to disappear, and the chlorine substitution at 6 positions will also lead to a significant decrease in titer. However, the substitution of tert-butyl isoxazole had the same activity as m-trifluoromethyl phenyl [IC_50_ (U937) = 0.63 μM]. The mode of action of these inhibitors was similar. The 1-aminoisoquinoline heterocycles were two-point hinge binder and formed hydrogen bonds with the main chain N and CO of residue Met 95, the urea group formed a hydrogen bond with the side chain of Glu63, and the carbonyl oxygen on the urea group formed a hydrogen bond with the amide on the main chain of Asp156 of the DLG motif (Harris et al., 2013), seen in Figure 4B. Compounds **1** and **8** (Figure 4A) showed high activity *in vitro*, but their activity in cells generally did not support the advancement of the series to *in vivo* models.

#### 4.2.2 Pyrrole [2,3-b]pyridines

**FIGURE 5:**
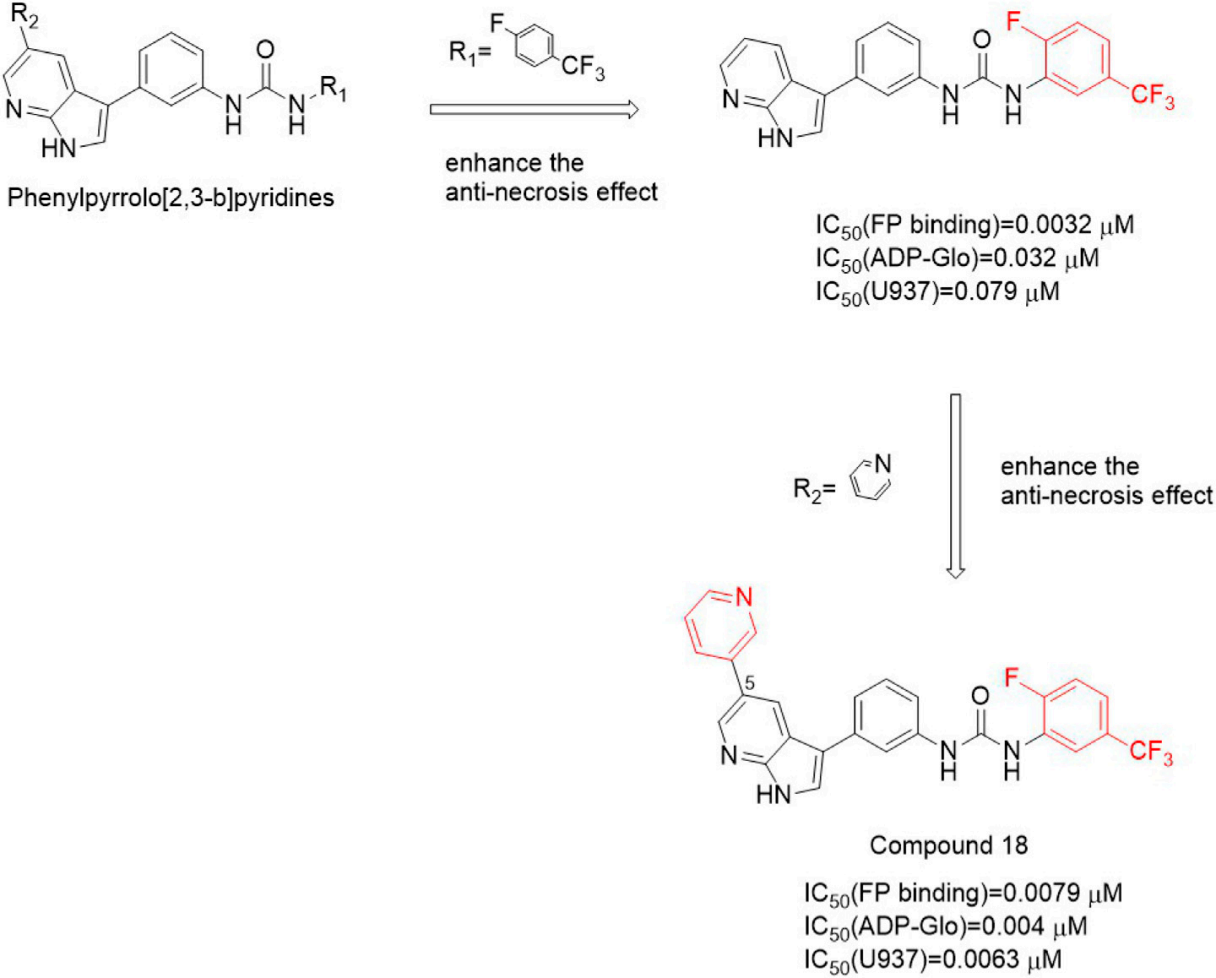
Structure of Compound **18** and it’s bioactivities.

These compounds used 5-phenylpyrrole [2-Phenylpyrrole]pyridine heterocycle as a hinge binder. The activity is greatly increased by small volume substituents attached to urea on the benzene ring. These small substituted groups existed in the lipophilic subbag of the DLG-out pocket, in which 3−trifluoromethyl substitution was the best [IC_50_ (FP binding) = 0.032 μM, IC_50_ (ADP-Glo) = 0.032 μM, IC_50_ (U937) = 0.079 μM], while cyclopentylurea substitution activity was weak. Compound **18** (Figure 5), in which the meta-pyridine group was substituted for the aromatic ring at the fifth position of the pyrrole [2,3-b]pyridine heterocyclic ring, exhibited good activity [IC_50_ (FP binding) = 0.0079 μM, IC_50_ (ADP-Glo) = 0.004 μM, IC_50_ (U937) = 0.0063 μM]. Although these compounds have good activity *in vitro*, the pharmacokinetic parameters are not good and are not suitable for *in vivo* models.

#### 4.2.3 Furo [2,3-d]pyrimidines

**FIGURE 6:**
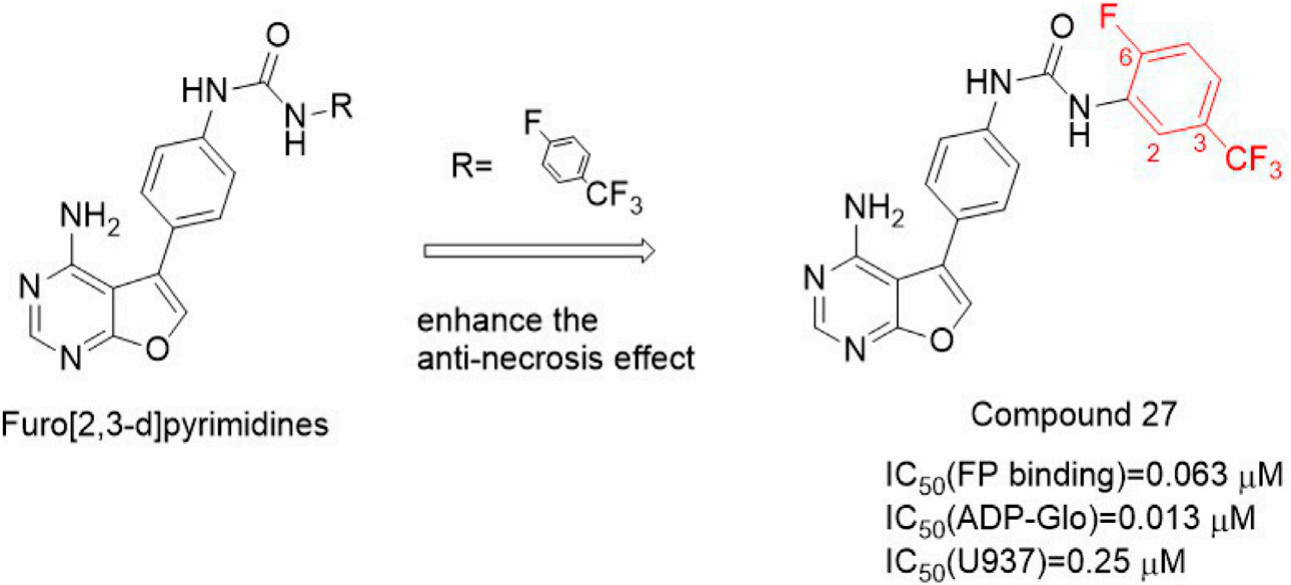
Structure of Compound **27** and it’s bioactivities.

The structure-activity relationship of this type of compound was similar to the previous two types. The structure-activity relationship around the urea group showed that the small group at the meta position had better substitution activity. The antinecrosis activity was decreased by replacing 2 or 6 positions of benzene ring substituents with fluorine atoms or chlorine atoms, and tert-butoxazole ring and 5-fluoro-3-(trifluoromethyl) phenyl groups were better. Due to the better pharmacokinetic performance of these inhibitors, the first type Ⅱ ATP enzyme inhibitor compound 27 [IC_50_ (FP-binding) = 0.063 μM] was obtained (Figure 6) and used in the mouse model of lethal shock induced by TNF-α. This compound had good oral exposure in mice [AUC = 14 ± 7 μg/h/ml and Cmax = 1,100 ng/ml, Male C57BL/6 mice were given compound **27** orally (2.0 mg/kg)] and plays a good role in hypothermia protection (Orozco et al., 2014)^.^

In 2013, Harris et al. (Harris et al., 2013) identified three type Ⅱ RIPK**1** kinase inhibitors, 1-aminoisoquinolines,pyrrole [2,3-b]pyridines, and furan [2,3-d]pyrimidines. All of them had good activity *in vitro*. However, the poor pharmacokinetics properties and off-target activity of these compounds limited their further research.

#### 4.2.4 PK6 and its derivatives

**FIGURE 7:**
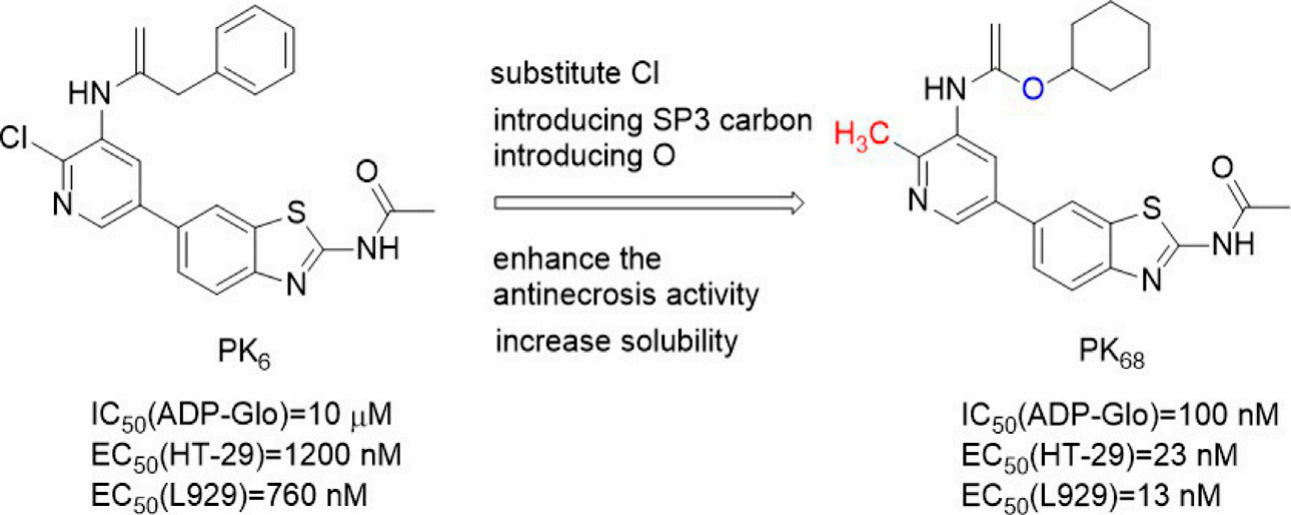
Structure of PK**6** and PK**68** and their bioactivities.

PK6 (Figure 7) effectively inhibited TNF-induced necrosis of mouse embryonic fibroblasts (MEF) and mouse fibroblast L929 cells with EC50 (L929) values of 0.95 μM and 0.76 μM, respectively. And PK6 inhibited TNF-induced necrosis of human leukemia U937 cells [EC_50_ (U937) = 1.33 μM] (Hou et al., 2019). Compared with PK**6** [IC_50_ (RIPK**1** Kinase activity) = 0.20 μM], the inhibitory activity of PK**68** (Figure 7) on RIPK**1** kinase was significantly enhanced, and its IC_50_ value was 90 nM, which was consistent with its anti-necrotic cell activity. At the same time, PK6 and PK68 did not affect the activity of RIPK**3** kinase. PK68 has reasonable selectivity and good pharmacokinetic properties for the inhibition of RIPK**1** kinase activity and has a strong protective effect on TNF-induced fatal shock *in vivo*. In addition, PK68 has also been observed to inhibit tumor metastasis in mouse cancer models of melanoma and lung cancer (Hou et al., 2019). As an effective and selective RIPK**1** inhibitor, PK**68** had great potential in the treatment of inflammatory diseases and cancer metastasis.

The 2-position chlorine atom on the pyridine ring of PK**6** was an active leaving group that caused toxicity. And PK**6**’s solubility was poor due to the number of sp2 carbon in PK6. PK**68** with higher anti-necrosis activity and better solubility was obtained by replacing chlorine atom with methyl and introducing SP3 carbon into the C ring, and at the same time introducing an oxygen atom into the top region of the scaffold. PK68 had EC_50_ (HT-29) values of 23 nM and EC_50_ (L929) values of 13 nM in human and mouse cells, respectively.

PK68 molecular docking with the RIPK**1** showed that PK**68** was Ⅱ type of RIPK**1** kinase inhibitors (Hou et al., 2019). PK**68** interacted with RIPK**1** protein motif of DLG, its N-acetamide and Met95 residues of the main chain CO formed hydrogen bonding, benzothiazole part formed hydrogen bonds with Ile43 residue, carbonyl oxygen of carbamic acid in PK**68** backbone formed hydrogen bonds with Asp156 residue of DLG motif, cyclohexane group buried deep in a hydrophobic allosteric bag (Hou et al., 2019). The bag contained RIPK**1** DLG-out conformation of residue Met66, Met67, Leu70, Val75, Leu129, Val134, and Leu159 (Hou et al., 2019).

**FIGURE 8:**
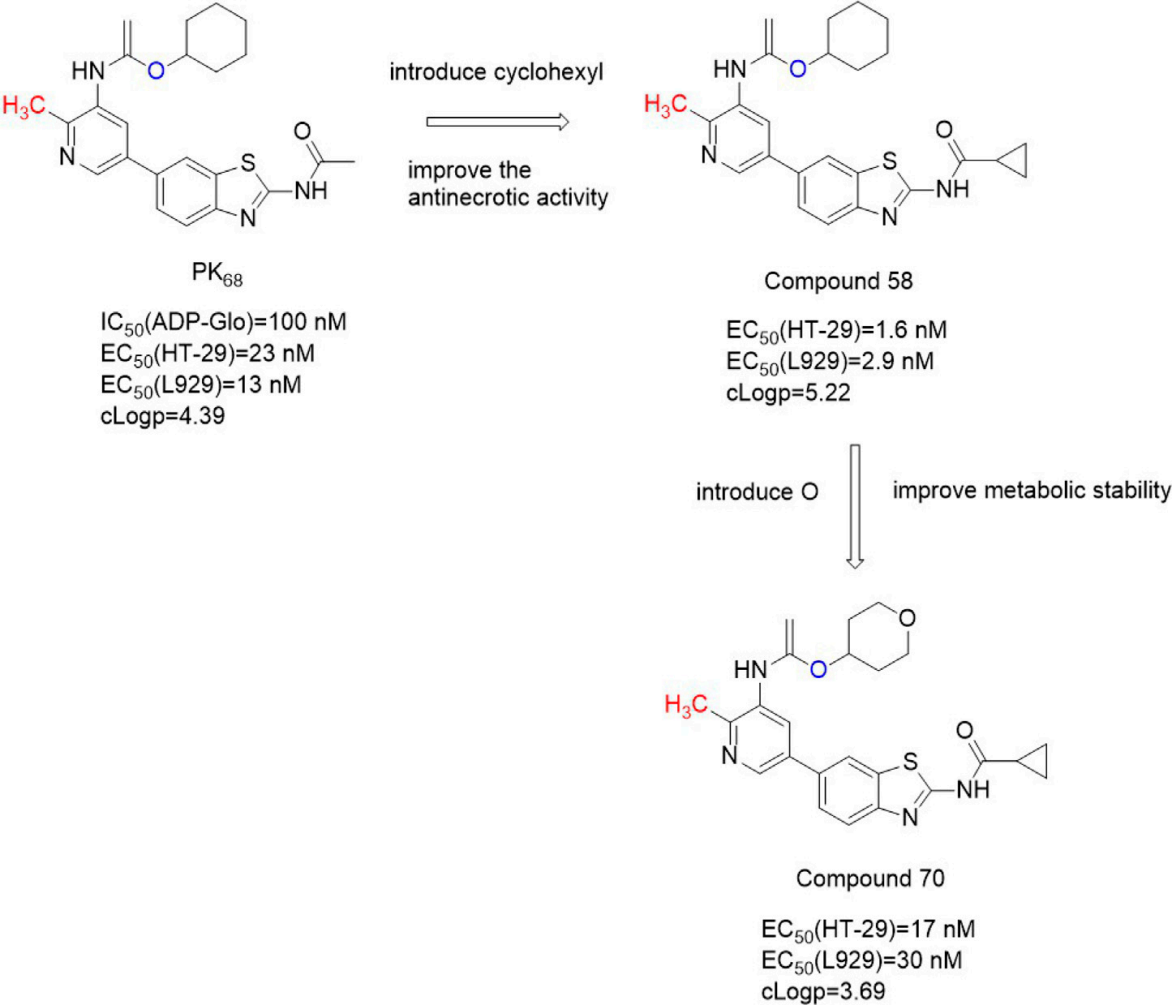
Structure of Compound **70** and it’s bioactivities.

The structure-activity relationship of PK**68** showed that the activity of the compound was negatively correlated with lipophilic activity. Removal or replacement of the acetyl group results in a decrease or loss of activity, possibly due to hydrogen-bonded interactions between N acetamide and Met 95. The R2 sites of pyridine and benzo [d]thiazole rings are exposed to solvents and can tolerate more structural diversity. Substitution of cyclopropyl at the R2 site significantly increased activity [EC_50_ (HT-29) = 1.6 nM, EC_50_ (L929)=2.9 nM)] (Li et al., 2022). There was an additional hydrogen bond between the amino acid nitrogen of Compound **58** and the Met92 residue of RIPK**1**, which significantly enhanced the effect of Compound **58** (Figure 8). However, Compound **58**'s clogP (5.22) was higher and Compound **58** had poor metabolic stability in human liver microsomes (Li et al., 2022). In order to reduce cLogP, cyclohexyl was replaced with a smaller aliphatic ring, resulting in a significant decrease in cLogP but also a decrease in anti-necrotic activity. By introducing oxygen atom into c-4 site of cyclohexane, Compound **70** (Figure 8) was obtained, which showed high binding affinity with RIPK**1** and significantly improved its metabolic stability in human liver microsomes (K_d_ = 9.2 ± 0.4 nM).

#### 4.2.5 GSK'074

**FIGURE 9:**
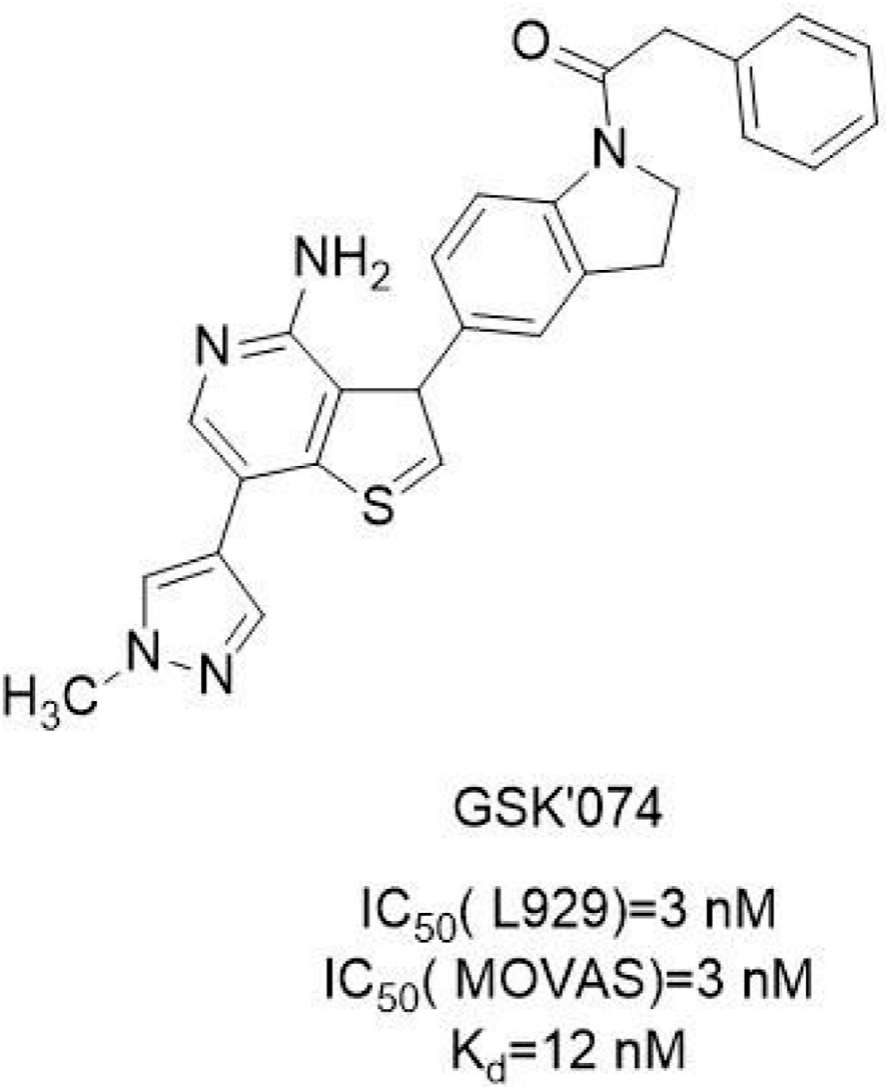
Structure of GSK'**074** and it’s bioactivities.

This type of new inhibitor represented by GSK2593074A (GSK'074) (Figure 9) is a dual inhibitor of RIPK**1** and RIPK**3**, which bound to RIPK**1** and RIPK**3** to inhibit the kinase activity of the two kinases. Meanwhile, GSK'**074** inhibited rip3-dependent necrosis and inflammation but RIPK**1**-independent. It could completely block the necrosis of human cells and mouse cells at 10 nM. The inhibition of RIPK**1** kinase activity by GSK ‘**074** at the concentration of 20 nmol/L was the same as that by 1 μM/L Nes-**1**s (Zhou et al., 2019).

The structure-activity relationship of GSK'**074** has not been reported. The molecular docking model showed that GSK'**074** bound well to DLG-out homology model of RIPK**1** and locked the kinase in an inactive conformation, suggesting that it was likely to be a type Ⅱ kinase inhibitor. GSK'**074** formed hydrogen bonds with the Glu93 and Met95 hinged backbone atoms in RIP2, while the pyrazole ring was oriented towards the solvent exposed region. In the DLG-out conformation, the benzyl part of GSK'**074** was connected to the hydrophobic pockets formed by Leu70, Val75, and Leu129 (Zhou et al., 2019).

#### 4.2.6 GEN684

**FIGURE 10:**
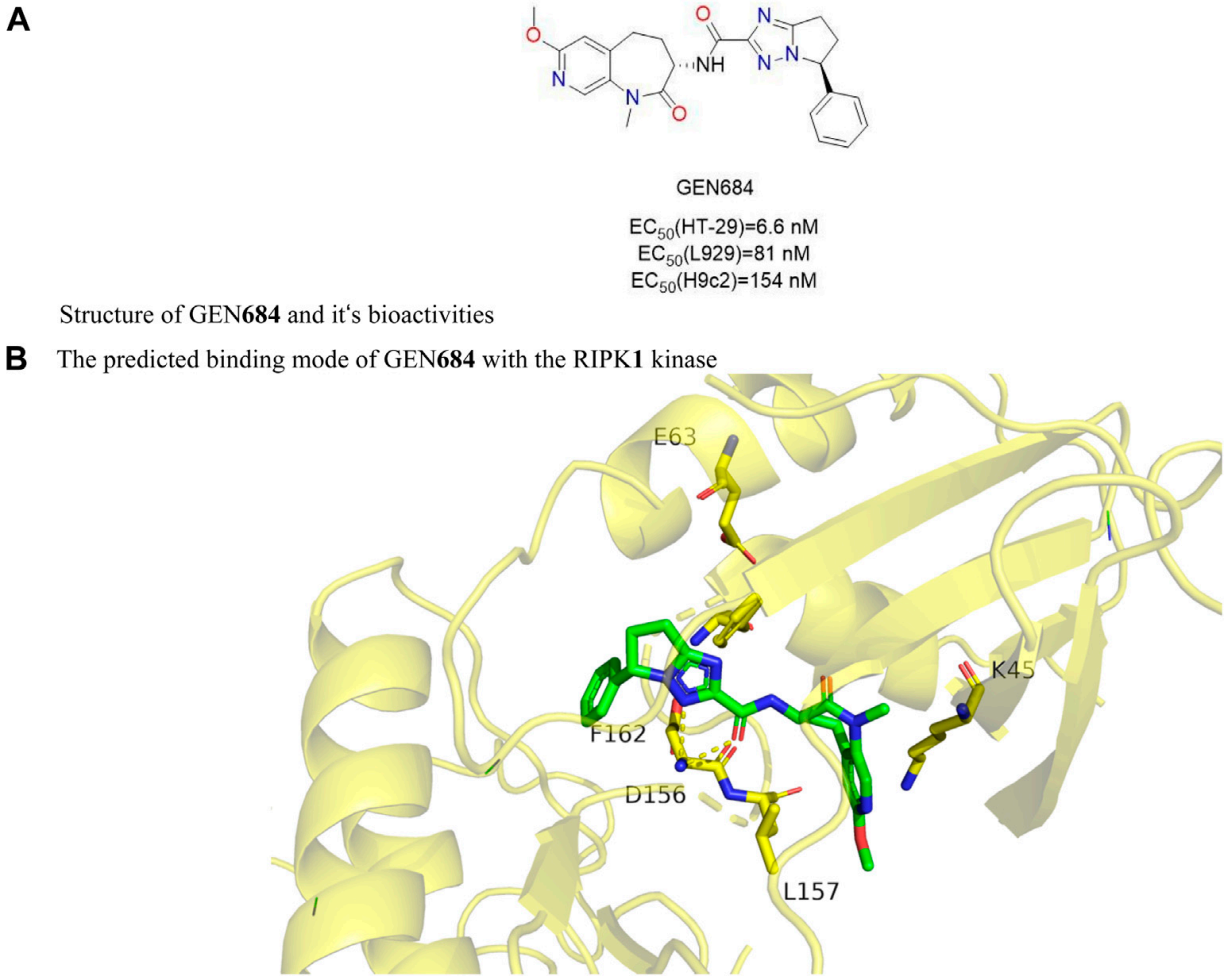
Structure of GEN**684** (PDB code: 6NYH) and it’s bioactivities and the binding mode of GEN**684** with the RIPK**1** kinase. **(A)**. Structure of GEN**684** and it‘s bioactivities. **(B)**. The predicted binding mode of GEN**684** with the RIPK**1** kinase.

GNE**684** (Figure 10A) i had a strong inhibitory effect on human RIPK**1** *in vitro* and a slightly weaker effect on mouse and rat RIPK**1**. The Ki values of RIPK**1** in humans, mice, and rats were 21 nM, 189 nM, and 691 nM, respectively. In three inflammatory disease models (TNF-driven SIRS, colitis caused by NEMO deficiency in inflammatory bowel disease, and collagen antibody-induced arthritis), GNE**684** played a protective role, suggesting that targeting RIPK**1** was effective in treating inflammatory diseases. However, in the PDAC model of KPP or KPR, GNE**684** did not affect on overall survival or tumor growth.

Structure-activity relationship of GNE**684** was unclear. The molecular docking model showed that GNE**684** bound to the inactive conformation of RIPK**1**, Asp156, and PHE162 of the DLG motif were in the “out” conformation, and the αC-helix was far from the ATP-binding fissure, Glu63 which catalyzed Lys45 and αC-helix lacked a typical ion pair (Patel et al., 2020).

### 4.3 Type Ⅲ kinase inhibitors

Unlike type II kinase inhibitors, type III kinase inhibitors have no hinge binding interaction and occupy an allosteric lipophilic pocket on the back of the ATP binding site, so the kinase selectivity is higher than that of type II kinase inhibitors.

#### 4.3.1 Indole hydantoin

**FIGURE 11:**
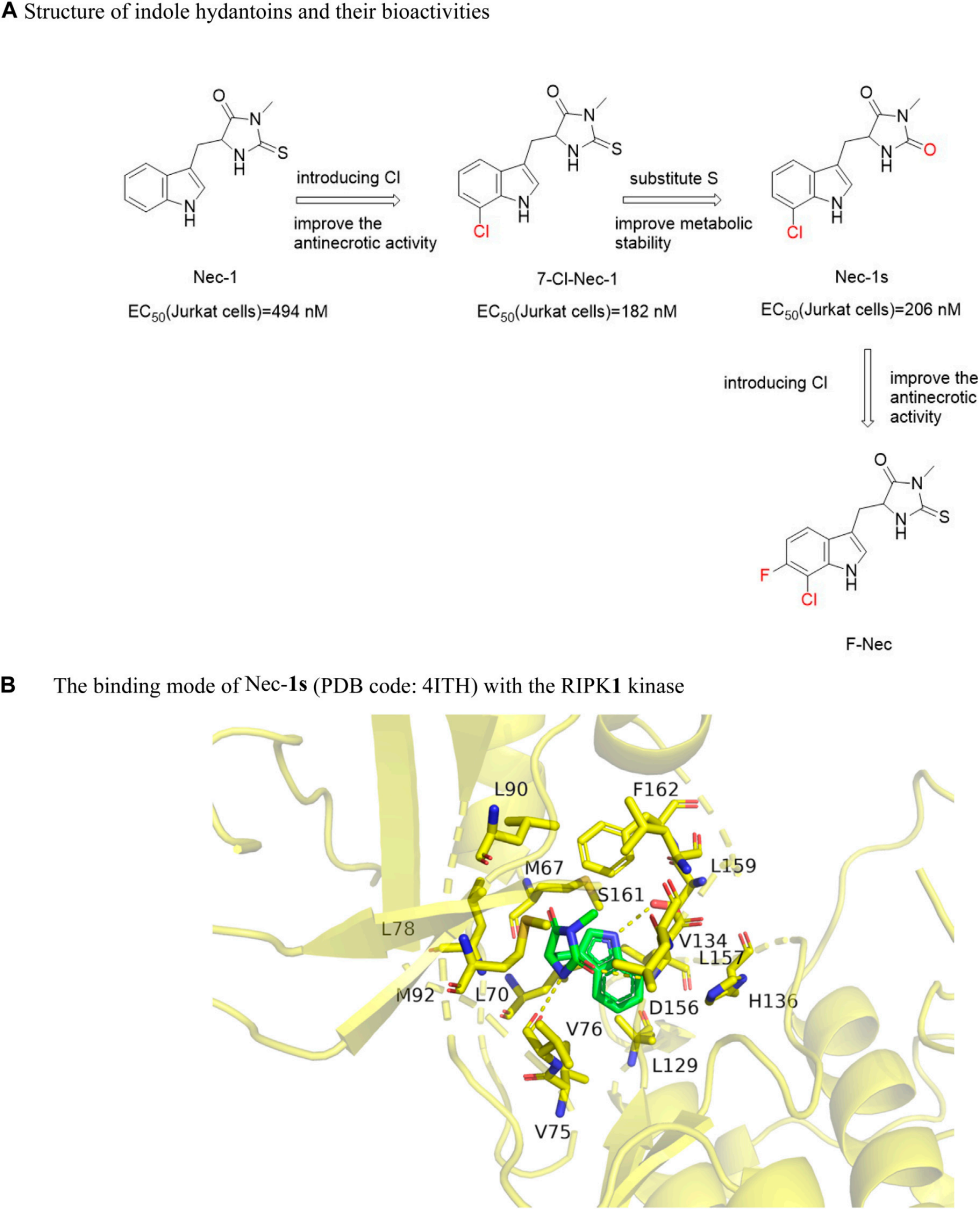
Structure of indole hydantoins and their bioactivities and the binding mode of ^Nec−**1s**^ (PDB code: 4ITH) with the RIPK**1** kinase. **(A)**. Structure of indole hydantoins and their bioactivities. **(B)**. The binding mode of ^Nec-**1s**^ (PDB code: 4ITH) with the RIPK**1** kinase.

Degterev et al. screened the first RIPK**1** kinase inhibitor Nec-**1** [EC_50_ (Jurkat) = 494 nM] (Figure 11A) that blocked the programmed necrosis of human monocyte U937 cells induced by TNF-α and zVAD.fmk from a chemical library containing 15,000 compounds. ^Nec−**1**^ improved the neurological function of mice after cerebral hemorrhage and reduced cerebral edema (Degterev et al., 2008). However, ^Nec−**1**^ was not a specific inhibitor of RIPK**1** because its chemical scaffold was similar to methyl-thiohydantoin-trytophan (MTH-Trp) (Takahashi et al., 2012). Structure-activity relationship analysis found that removing the methyl group on the hydantoin would eliminate its anti-necrotic activity. Nec-1 has poor metabolic stability *in vivo* and targeted indoleamine-2,3-dioxygenase (IDO). Adding chlorine (7-Cl-NEC-1) to the benzene ring of Nec-1, the activity increased about 2.7 times [(EC_50_ (Jurkat) = 182 nM]. Then the sulfur in the hydantoin was replaced with oxygen to obtain Nec-**1**s [7 -Cl-O-Nec-1; EC_50_ (Jurkat) = 206 nM)] (Figure 11A), the anti-necrotic activity was not affected and it was more stable in metabolism.

By introducing fluorine atoms into the A ring of NEC-**1**, F-Nec was obtained. F-Nec showed high anti-necrosis activity, and the inhibitory activity of F-Nec was more than 8 times that of NEC-**1**. In animal experiments, F-Nec can effectively improve the activation of necrotizing ptosis induced by endotoxin/galactose in mice, and reduce liver injury and inflammation (Li et al., 2021). F-Nec may be a candidate compound for the development of inflammatory diseases caused by necrotizing ptosis.

The crystal structure analysis of Nec-**1**s and RIPK**1** showed that Nec-**1**s was buried in a L-shaped hydrophobic bag between the N-terminal and C-terminal. The indole ring interacted with six amino acids Met67, Leu70, Val75, Leu129, Val134, and His136 through van der Waals force, while the five-membered ring was encompassed by hydrophobic amino acids Val76, Leu78, Leu90, Met92, Leu157, Leu159, and Phe162 (Xie et al., 2013). These hydrophobic interactions were the main driving force for the binding. In addition to the hydrophobic interaction, hydrogen bonds were formed between the nitrogen atoms on the indole ring and the hydroxyl oxygen of Ser161 on A-Loop, and the five-membered ring formed hydrogen bonds with the carbonyl oxygen of Val76 and amide nitrogen of Asp156 (Xie et al., 2013) (Figure 11B). These three hydrogen bonds lock the orientation of ^Nec−**1**s^ into the oil cavity of RIPK**1**. Considering the potency and selectivity of these inhibitors to inhibit RIPK**1** kinase, they could serve as a useful probe for RIPK**1** kinase activity *in vitro* and *in vivo* (Degterev et al., 2008).

**FIGURE 12:**
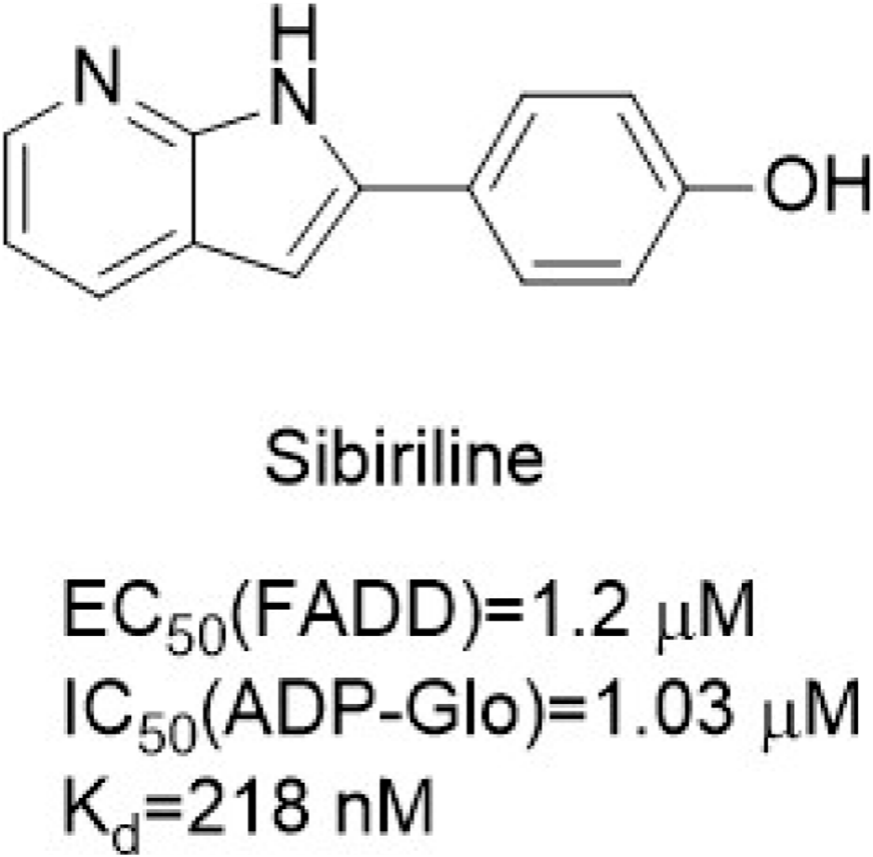
Structure of Sibiriline and it’s bioactivities.

Sibiriline (Figure 12) was a novel pyridine derivative based on pyrrole [2,3-b]pyridine screened from a small molecule compound library. Sibiriline inhibited TNF-induced RIPK**1**-dependent necrosis [EC_50_ (FADD) = 1.2 μM] in FADD-deficient Jurkat cells, had a high affinity for RIPK**1** (Kd = 218 nM), and effectively inhibited the phosphorylation level of phosphorylated myelin basic protein (MBP) [IC_50_ (ADP-Glo) = 1.03 μM]. Sibiriline had a protective effect on immune-mediated acute hepatitis in mice by significantly reducing the level of aspartate aminotransferase (AST)/alanine aminotransferase (ALT) and liver injury. However, sibiriline is only moderately specific, as 104 interactions have been identified.

Sibiriline has a similar chemical structure to Pyrrole [2,3-b]pyridinesSibiriline, but its binding pattern is the same as Nec-**1**. Sibiriline is bound to the DLG motif and gatekeeper in the “back” pocket of RIPK**1** (Najjar et al., 2015; Fabienne, 2017). RIPK**1** was locked in the kinase in the DLG-out conformation by generating hydrogen bonds with Asp156 of the DLG motif and adjacent Ser161, and interacted with α-C helix, but did not interact with the hinge region (Fabienne, 2017).

#### 4.3.2 Phenylbutyramides

**FIGURE 13:**
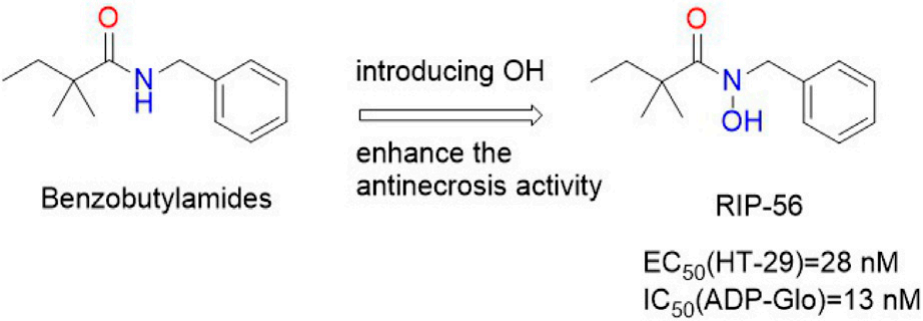
Structure of RIP-**56** and it’s bioactivities.

Yan Ren et al. screened a new class of amide-containing lead compounds from the chemical library of 200,000 compounds by TSZ-induced HT-29 cell necrosis test and obtained RIPA-**56** through reasonable structure-activity relationship optimizatio. RIPA-**56** (Figure 13) was similar to the molecular docking model of Nec-**1**s by inhibiting RIPK**1** kinase in its inactive form. The activity of RIPK**1** kinase was effectively inhibited by RIPA-**56**, and its IC_50_ value was 13 nM. And RIPA-**56** had no inhibitory effect on the activity of RIPK**3** kinase. RIPA-**56** also showed a protective effect on mouse L929 cells from TZS-induced necrosis [EC_50_ (L929) = 27 nM].

The molecular docking model between RIPA-**56** crystal and RIPK**1** showed that the carbonyl oxygen on the benzene ring of RIPA-**56** formed a hydrogen bond with the main chain NH on Asp**156** of RIPK**1**, and 2,2-dimethylbutanamide formed a hydrogen bond with Val76 of RIPK**1**, so that RIPA-**56** tightly bound to the L-shaped hydrophobic pocket of RIPK**1** (Ren et al., 2017). The benzene ring occupied the left hydrophobic pocket formed by Leu70, Leu129, Val134, His136, Ile154, and Ser161. The narrow space of this pocket did not tolerate benzene rings or polar aromatic heterocycles with larger substituents. The introduction of fluorine atoms into the benzene ring increased the anti-necrotic activity, while the introduction of larger groups such as bromine or chlorine led to the loss of anti-necrotic activity. The titer of 2,3, 5-trifluoro substituents was higher and the antinecrotic actvity of 2,4, 6-trifluoro substituents was significantly decreased. In addition, the introduction of methyl on the nitrogen atom of the amide could increase the potency by about 10–40 times, and the introduction of a larger ethyl group would completely lose the potency due to steric hindrance. So the smaller hydroxyl group was introduced. The hydrophobic pocket on the right was sensitive to the size and polarity of the substituted acyl group. Adding one carbon and the lack of one carbon significantly reduced the effectiveness. It was found that only 2, 2-dimethylbutanamide groups could effectively occupy this hydrophobic pocket space.

#### 4.3.3 Benzoxazepines

**FIGURE 14:**
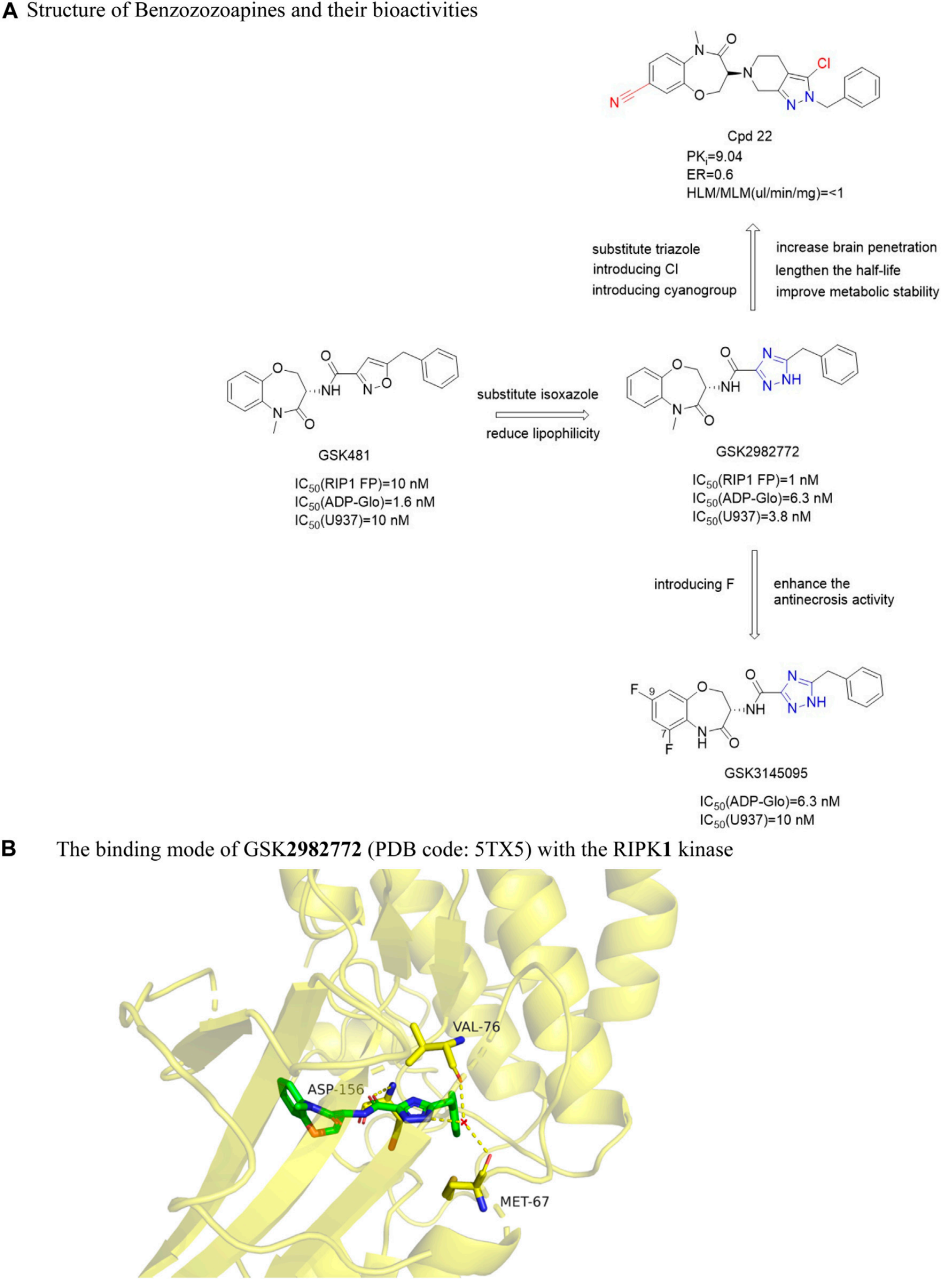
Structure of Benzozozoapines and their bioactivities and the binding mode of GSK**2982772** (PDB code: 5TX5) with the RIPK**1** kinase. **(A)**. Structure of Benzozozoapines and their bioactivities. **(B)**. The binding mode of GSK**2982772** (PDB code: 5TX5) with the RIPK**1** kinase.

Harris et al. (Harris et al., 2016) found that benzoxazepines had strong biochemical activity through DNA encoding small molecule libraries. Firstly, it was discovered that GSK'**481** (Figure 14A) has an IC_50_ (RIPK**1** FP) value of 10 nM, which inhibited the phosphorylation of Ser166 in wild-type human RIPK**1** [IC_50_ (Human WT) = 2.8 nM]. However, GSK'481 had high lipophilicity and sub-optimal pharmacokinetic properties (Harris et al., 2017). Then the group optimized GSK'481 to obtain GSK**2982772** (Figure 14A). The IC_50_ (RIPK**1** FP) against RIPK**1** was 1.0 nM *in vitro*. The compound had good activity and pharmacokinetic properties and significantly avoided hypothermia in mice in the inflammatory response model induced by TNF-α. Further optimization found that GSK**3145095** (Figure 14A) had an *in vitro* IC_50_ (ADP-Glo) value of 6.3 nM for RIPK**1**. Benzooxazepines had good selectivity to RIPK**1** and unique species selectivity to primate and non-primate RIPK**1**. In addition, Yoshikawa et al. found Cpd**22** (Figure 14A), an oral cerebral transbenzozozoapine RIPK**1** kinase inhibitor with good pharmacokinetic activity, significantly inhibited programmed necrosis in human colon cancer cells HT-29 [IC_50_ (HT-29) = 2.0 nM] and mouse fibroblasts L929 [IC_50_ (L929) = 15 nM] in response to the low brain tissue distribution of the inhibitor GSK**298277259** (Yoshikawa et al., 2018).

The eutectic structure of GSK**2982772** and RIPK**1** showed that the benzooxazine ring was deeply buried in the pocket between the N-terminal and C-terminal domain, and the triazole ring and benzyl groups occupied the allosteric lipophilic pocket on the back of the ATP binding site, so that the inhibitor was located deeper in the ATP binding pocket (Harris et al., 2017). The amide carbonyl group of the triazole ring interacted with the main chain amide NH of Asp156, and triazole formed a hydrogen bond with the carbonyl oxygen of Met67 and Val76 (Harris et al., 2017). The binding mode of GSK**3145095** and RIPK**1** was similar to GSK**2982772**, excepted that there was a hydrogen bond between the lactam nitrogen of GSK**2982772** and the main chain carbonyl group of Leu90, and the fluorine at the 9-position of the benzoxazepine ring was located between Met92 and Ile43 (Harris et al., 2019a) (Figure 14B).

The benzoxazepine ring was located in a narrow pocket by the formation of two β sheets Leu90-Val91-Met92 and Ile43-Met44-Lys45. Increasing the size of the lactam nitrogen or chiral center or changing the conformation of the 7-membered ring could reduce the effectiveness. The 7 and 8-position substitutions on the aromatic ring are well tolerated because they are oriented towards the solvent-exposed region. Only the titer of fluorine substitution at 6 and nine sites was better. Although the methylation of lactam increased the effectiveness of RIPK**1** by 10 times, it also increased the liver metabolic rate of the compound. Keeping the original lactam group NH had better exposure *in vivo*. Replacing the isoxazole ring with a triazole ring did not change the efficacy of RIPK**1** and significantly reduces lipophilicity. The heterocyclic “unbridged” nitrogen ortho with the amide carbonyl group had the best potency due to the electron lone pair repulsion separating the negatively charged nitrogen from the amide oxygen atom. This nitrogen atom near the carbonyl group gave a favorable trans orientation, thus allowing the benzyl group to be optimally located in the narrow back pocket. The 7, 9-difluorine substitution on the benzozoazapine rim of GSK**3145095** increased its potency due to the fact that the electron-withdrawing fluorine atom reduced the pKa of nitrogen on the lactam, thereby enhancing its hydrogen bonding with the Leu90 mainchain.

In order to increase the brain permeability, on the basis of GSK**2982772**, the 6,5 bicyclic core structure was replaced by 4-oxy-6, 7-dihydro-1H-imidazole [4, 5-c] pyridine ring, which led to a significant decrease in p-glycoprotein-mediated efflux and an enhancement of binding affinity. The introduction of lipophilic group and polar group into pyrazole ring significantly enhanced the binding affinity, and the introduction of chlorine atom was the best, which could significantly prolong the half-life. The benzene ring on the benzoxazine ring had metabolic instability because of its high electron density. If the electron-absorbing substituted cyano group was introduced at positions seven and eight of the benzoxazine, exposure of the cyano group to the solvent accessible region could reduce the electron density of benzoxazinone and improve the metabolic ability. The obtained Cpd**22** inhibited the death and phosphorylation of necrotic cells in HT-29 and L929 cells. And *in vivo* studies have shown that Cpd**22** can delay the disease progression of autoimmune encephalomyelitis (EAE) model mice (Yoshikawa et al., 2018). These inhibitors can be used as central nervous system tool compounds for the study of RIPK**1** kinase.

**FIGURE 15:**
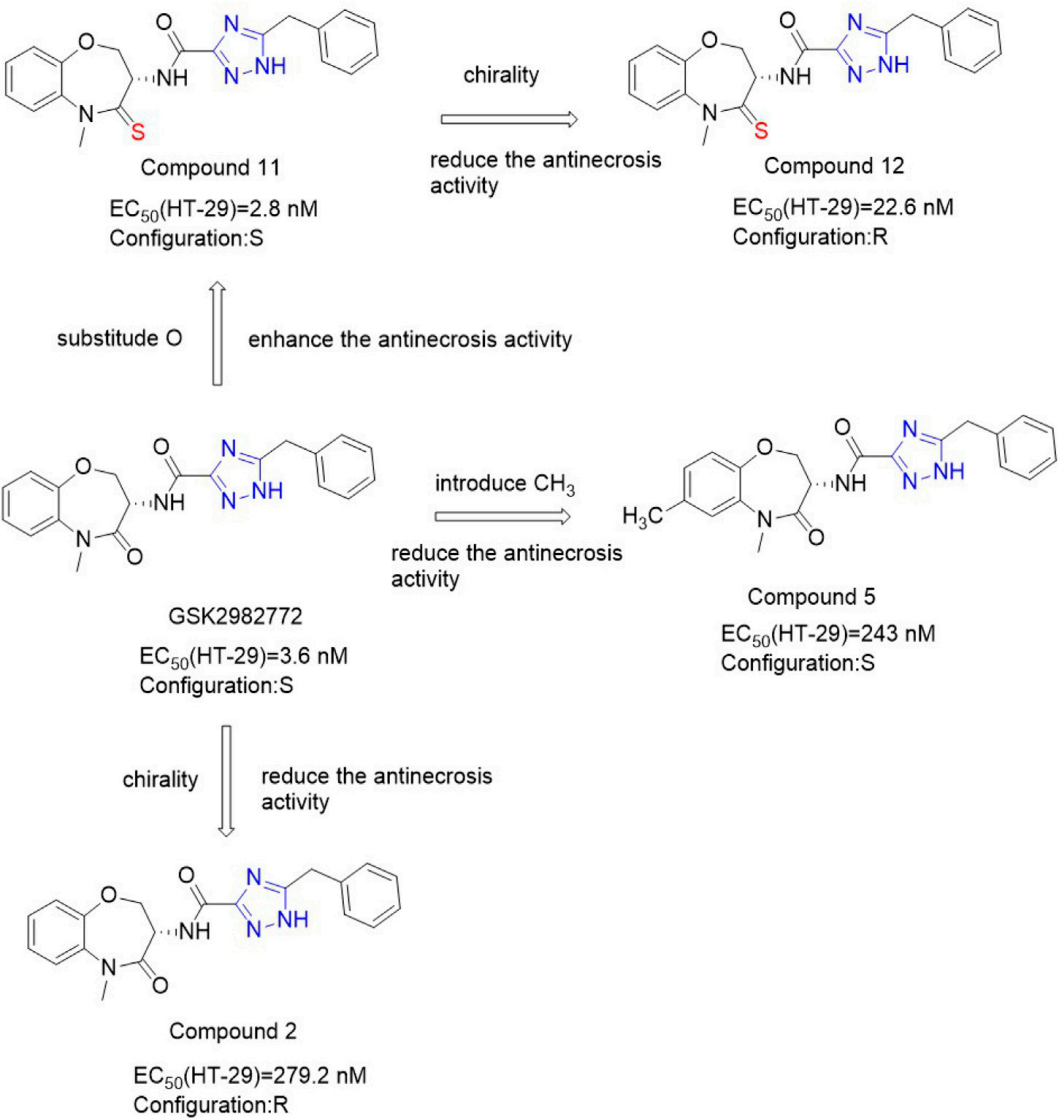
Structure of Benzoxazepines and Thio-benzoxazepinones and their bioactivities.

Because the benzene ring is not attached to the hinge region, this position can be replaced by a heterocyclic ring by a bioisosterism. When benzene ring is replaced by different pyridine, the anti-necrosis activity decreases. The anti-necrosis activity of S configuration compounds with a methyl group added to the pyridine ring was improved [EC_50_ (HT-29) = 243 nM] (Xia et al., 2021). Chiral benzooxacyclic ketones exhibit distinct configurational activity. GSK**2982772** with S configuration has more than 70 times more anti-necrotizing droop activity than compound **2** (Figure 15) with R configuration. Since the carbonyl group of benzo azone fragment does not have any hydrogen bonding with surrounding residues, it can be used as a structural modification site. Thio-benoxazyclic ketones are obtained by replacing the oxygen atom on the carbonyl group with a sulfur atom. Since longer C=S bond lengths than C=O lead to greater steric hindrance, compounds containing C=O are more flexible and have higher bond angles than confined C=S compounds (Xia et al., 2021). This results in Compound **11** (Figure 15) and **12** (Figure 15) exhibiting similar conformations, resulting in less difference in their activity. Compounds **11** and **12** maintained high anti-necrosis activity in s-type necrosis model of human HT-29 cells. Based on this, thiobenoxazacyclic ketones may be the lead compounds to discover more inhibitors for the further development of necrotizing ptosis related diseases.

#### 4.3.4 GSK'547

**FIGURE 16:**
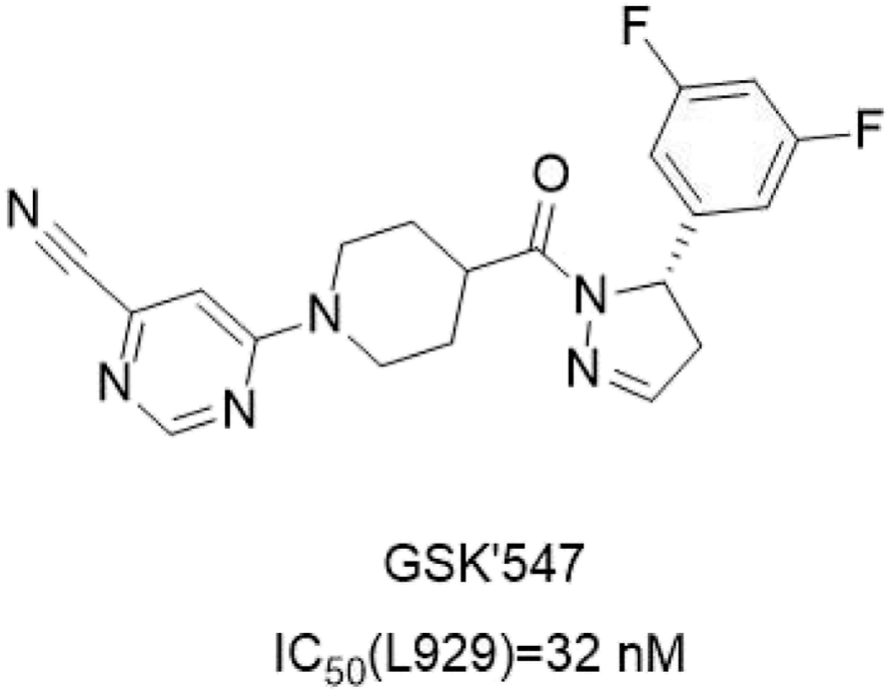
Structure of GSK′**547** and it’s bioactivities.

GSK'**547** (Figure 16) was an analog of GSK'963. Compared with GSK'963, GSK'**547** showed a 400-fold improvement in oral pharmacokinetic in mice. The structure-activity relationship of GSK’**547** has not been reported yet. The co-crystallization of GSK′**547** in the kinase structure fragment of RIPK**1** indicated that RIPK**1** binds in the allosteric pocket between the N-terminal and C-terminal domains behind the ATP binding site (Wang L. et al., 2018). In mouse models of pancreatic cancer tumors, GSK′**547** reduced tumor size and activated immune cell responses. And the drug molecule could be combined with PD-1 inhibitors for the treatment of pancreatic cancer. Although GSK′**547** was an excellent tool for exploring RIPK**1** inhibition in mouse tumor models, its high turnover rate in human hepatocytes did not possess the necessary characteristics as a clinical lead compound. Although GSK′**547** is used to investigate the inhibitory effect of RIPK**1** in mouse tumor models, its high conversion rate in human liver cells prevents it from being used as a clinical lead compound.

#### 4.3.5 Dihydropyrazoles

**FIGURE 17:**
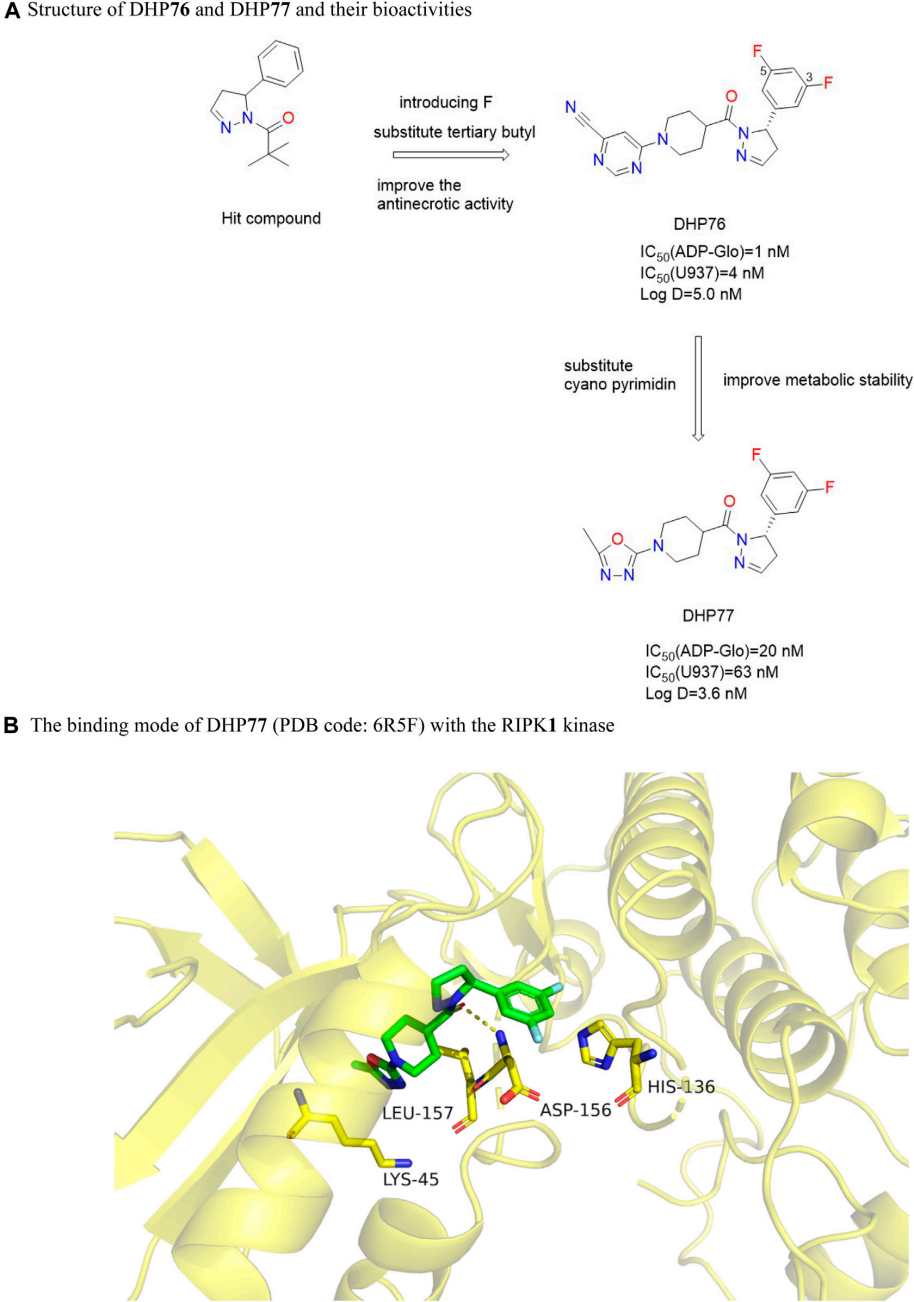
Structure of DHP**76** and DHP**77** and their bioactivities and the binding mode of DHP**77** (PDB code: 6R5F) with the RIPK**1** kinase. **(A)**. Structure of DHP**76** and DHP**77** and their bioactivities. **(B)**. The binding mode of DHP**77** (PDB code: 6R5F) with the RIPK**1** kinase.

Such compounds were obtained by high-throughput screening of the GSK compound library. DHP**76** (Figure 17A) performed well in the RIPK**1** FP/ADP-Glo binding experiment with an IC_50_ (ADP-Glo) value of 1.0 nM, which inhibited TNF-induced necrosis in L929 cells, with an IC_50_ (L929) value of 4.0 nM (Harris et al., 2019b). DHP**77** (Figure 17A) had good pharmacokinetic characteristics in a variety of species. Meanwhile, DHP**77** had good pharmacokinetic stability in rat and human hepatocytes and well predicted the pharmacokinetic parameters in humans. DHP**76** was effective in chronic mouse models of multiple sclerosis (EAE) and retinitis pigmentosa (Rd10).

Adding methyl to the dihydropyrazole heterocyclic ring or turning it into a benzene ring led to a decrease in activity. Replacing the dihydropyrazole heterocyclic ring with a six-membered ring or a thiazoline ring also significantly decreases the activity. This was due to changes in the central ring leading to conformational changes in the aryl and amide groups, which were not conducive to inhibitor binding. The benzene ring around the dihydropyrazole heterocycle tolerated smaller lipophilic groups, but larger substituents such as tert-butyl or larger polar groups were disadvantageous to the efficacy. The potency of U937 cell activity was moderately increased by adding 3,5-difluoro substitute to the benzene ring, because the fluorine atom provided additional van der Waals force at the aryl ring binding at the back of the allosteric pocket. Reducing the lipophilicity of these compounds lowered their clearance rate in the particles. Since the N-acetylpiperidol group points to the opening of the allosteric binding pocket, reducing its size or adding heteroatoms reduces potency, but adding substituents to the N-acetylpiperidol ring improves stability in rat and human liver microsomes. The substitution of nitrogen atoms in piperidine ring with cyano-containing heterocyclic ring had excellent cell activity but poor stability, while the substitution of stable 5-methyl-1,3, 4-oxadiazole ring could improve cell potency and stability.

DHP77 is bound to the allosteric region behind the ATP pocket. Although DHP77 and the adenine ring of ATP did not occupy the same space, the piperidine oxadiazole part occupied the space where ATP phosphate was located, and DHP77 exhibited a competitive inhibitory effect on ATP (Harris et al., 2019a). The pyrazole carbonyl of DHP77 received hydrogen bonds from the nitrogen of the main chain of Asp156 (Harris et al., 2019b). The chair conformation of the piperidine ring showed a complementary shape to the pocket geometry and provided a carrier for the oxadiazole ring to enter the solvation front at the pocket entrance of the active site between Lys45 and Leu157 (Harris et al., 2019a) (Figure 17B).

#### 4.3.6 2-Aminobenzimidazoles

**FIGURE 18:**
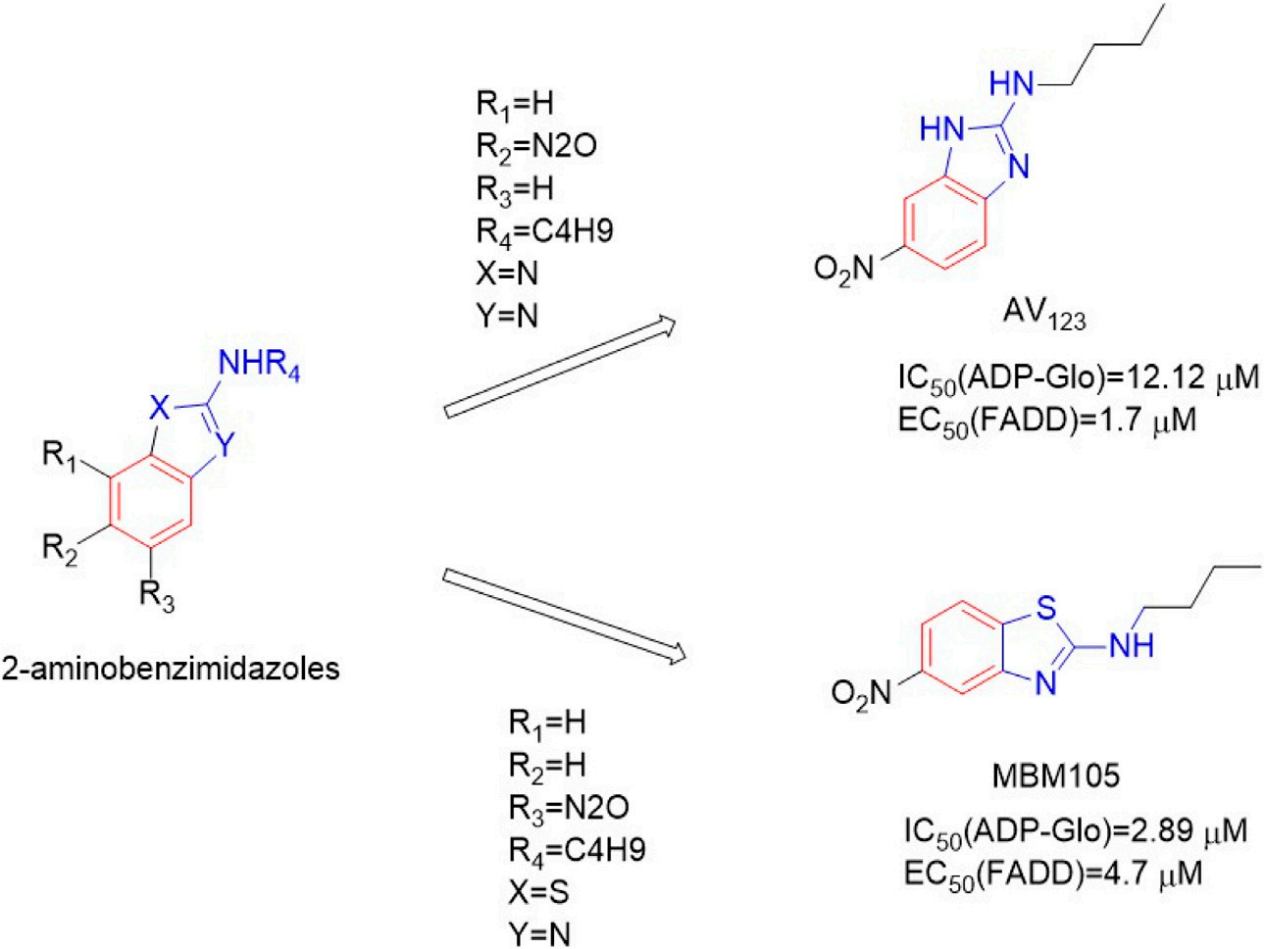
Structure of AV**123** and MBM**105** and their bioactivities.

This series of compounds was based on 2-aminobenzimidazole as the backbone (Harris et al., 2019b). 2-Aminobenzimidazole AV**123** (Figure 18) and 2-aminobenzothiazole MBM**105** (Figure 18) significantly blocked TNF-α-induced necrotic cell death in human FADD-deficient Jurkat cells, with EC_50_ (FADD) values of 1.7μM and 4.7 μM, respectively. Among them, MBM**105** had the best inhibitory effect on RIPK**1**, with an IC_50_ (ADP-Glo) value of 2.89 μM AV**123** and MBM**105** effectively inhibited the phosphorylation of MBP by RIPK**1**, with IC_50_ (ADP-Glo) values of 12.12 and 2.89 μM, respectively, in a dose-dependent manner. These compounds also had good selectivity for 12 disease-related protein kinases. *In vitro* studies on human RPE1 retinal cells and Jurkat wild-type lymphocytes confirmed that it can prevent necrotic cell death, but not apoptotic cell death.

The structure-activity relationship showed that the R1, R2, R3 and R4 positions of the benzimidazole nucleus only tolerated a small amount of modification. The amino group in AV123 had the best activity of n-butyl substitution. In addition, replacing the 2-aminobenzimidazole ring with the 2-aminobenzothiazole ring was conducive to the anti-ciliary activity, resulting in MBM**105**.

AV**123** formed hydrogen bonds with Asp156 main chain NH through N-3, the NH-nBu substituents formed hydrophobic poules with the side chains of residues Leu70, Val75, Ile154, Leu129, Val134, PHE162, Ser161, HiS136, and Asp156 to form hydrophobic interaction (Harris et al., 2019a). The hydrophobic interaction controlled the overall orientation of AV**123** at the binding site and located the nitrogroup between Leu90 and Met92 (Benchekroun et al., 2020). The same binding pattern was observed in MBM**105** (Benchekroun et al., 2020).

### 4.4 Others

#### 4.4.1 ZB-R-55

**FIGURE 19:**
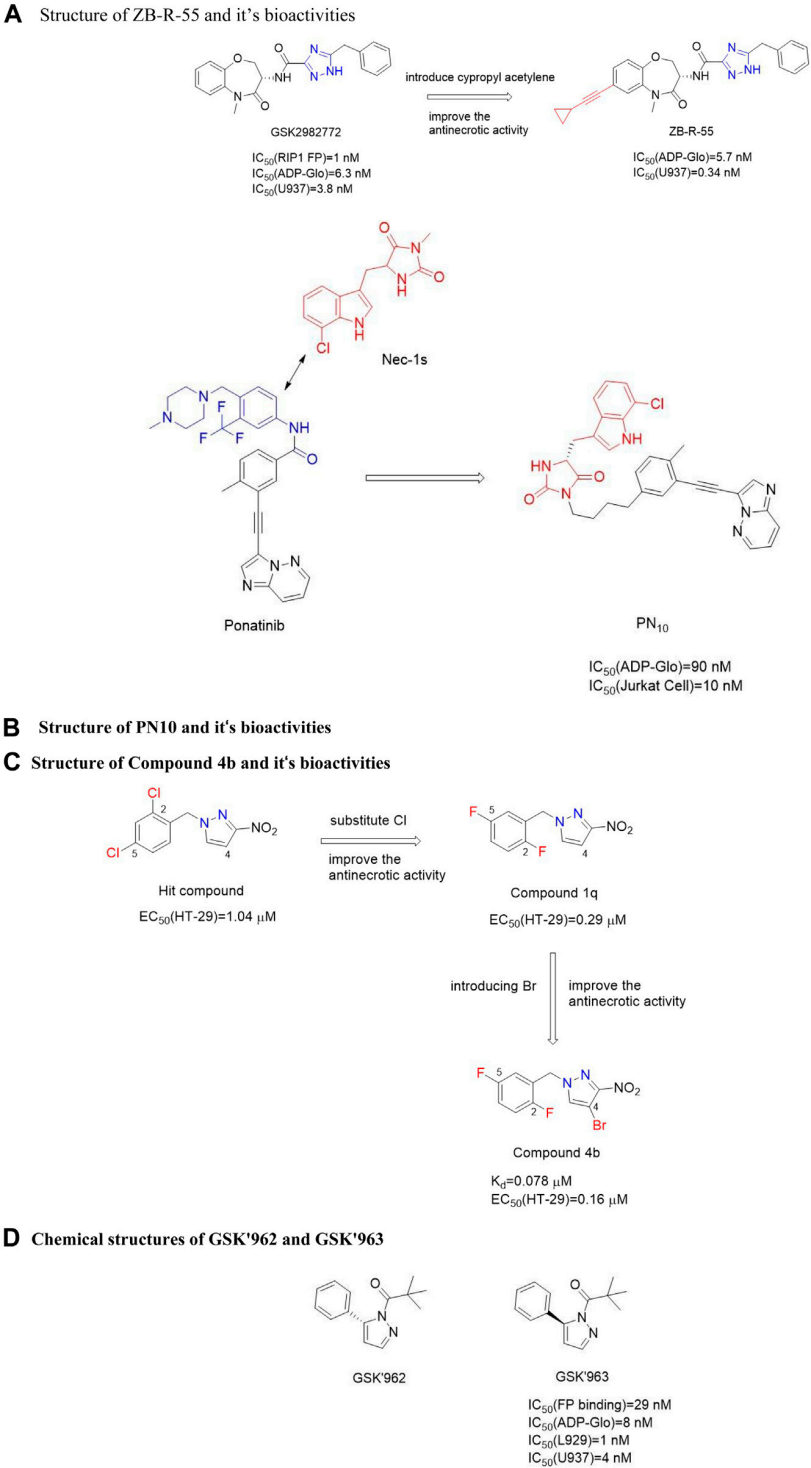
Structure of others and their bioactivities. **(A)**. Structure of ZB-R-55 and it’s bioactivities. **(B)**. Structure of PN10 and it’s bioactivities. **(C)**. Structure of Compound 4b and it‘s bioactivities. **(D)**. Chemical structures of GSK'962 and GSK'963.

ZB-R-55 (Figure 19A) is a novel dual mode RIPK1 inhibitor targeting both allosteric and ATP-binding pockets. Benzodiazepine introduced alkynyl groups could be inserted into the ATP-binding pocket, and aromatic or aliphatic substitutions introduced into the alkynyl groups could bind to key residues in the pocket for additional hydrophobic or electrostatic interactions. Benzyl was installed in allosteric pockets and hydrophobic with the side chains of M67, L70, L129, S161 and H136 (Yang et al., 2022). Triazoles form hydrogen bonds with main chains M67, V76 and L78 (Yang et al., 2022). In addition, the amide carbonyl group and triazole form hydrogen bonds with the amide main chain NH of D156, and the benzoxazine part forms hydrophobic interactions with the side chains of M92 and L157 (Yang et al., 2022). Cypropyl acetylene is introduced on the alkyne group of 21 into the ATP binding pocket and produces additional hydrophobicity with S25, V31, G98 and L157. *In vivo* pharmacokinetics, ZB-R-55 showed good pharmacokinetic characteristics [AUC = 15.018 μg/h/ml and C_max_ = 3,423 ng/ml, mice were given ZB-R-55 orally (3.0 mg/kg)]. ZB-R-55 has good *in vitro* anti-necrotizing droop and pharmacokinetic properties, which can be used to evaluate the efficacy of systemic inflammatory response syndrome (SIRS) model and lipopolysaccharide-induced sepsis model *in vivo*. Moreover, ZB-R-55 showed similar efficacy to glucocorticoid dexamethason in inhibiting cytokine storm (Yang et al., 2022).

**FIGURE 20:**
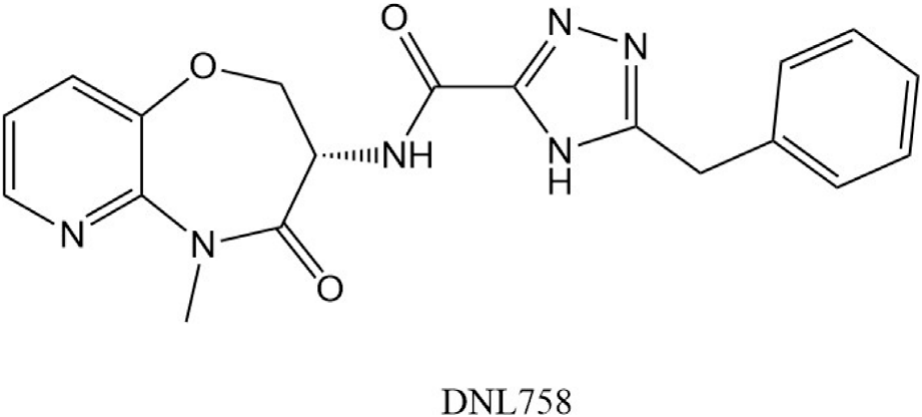
Chemical structure of DNL**758**.

**TABLE 1:** RIPK**1** inhibitors in clinical trial.

Inhibitor	Company	Disease type	Clinical research	ClinicalTrials.gov identifier
GSK**2982772**	GlaxoSmithKline	Psoriasis, rheumatoid arthritis and ulcerative colitis	Phase Ⅰ/Ⅱ	NCT04316585^1^
				NCT02903966^2^
				NCT03590613^3^
				NCT03305419^4^
				NCT02776033^5^
GSK**3145095**	GlaxoSmithKline	Single drug for the treatment of advanced or metastatic pancreatic ductal adenocarcinoma; combined with pembrolizumab (K or other anticancer drugs for the treatment of pancreatic ductal adenocarcinoma, non-small cell lung cancer, triple negative breast cancer, melanoma and other solid tumors	Phase Ⅱ(terminated)	NCT03681951^6^
DNL**747**	Denali Therapeutics	Alzheimer’s Disease	discontinue	NCT03757325^7^
DNL**758**	Denali Therapeutics	Cutaneous Lupus Erythematosus	Phase Ⅱ	Undisclosed
R**552**	Rigel	Central Nervous System Diseases	Phase Ⅰ	Undisclosed

^1^ClinicalTrials.gov. Study Record Detail. https://clinicaltrials.gov/ct2/show/NCT04316585?cond=GSK2982772&draw=2&rank=1 (accessed 18 July 2022). Reference to a dataset [dataset], 2022, (accessed 18 July 2022). ^2^ClinicalTrials.gov. Study Record Detail. https://clinicaltrials.gov/ct2/show/NCT02903966?cond=GSK2982772&draw=2&rank=2 (accessed 18 July 2022). Reference to a dataset [dataset], 2022, (accessed 18 July 2022). ^3^ClinicalTrials.gov. Study Record Detail. https://clinicaltrials.gov/ct2/show/NCT03590613?cond=GSK2982772&draw=2&rank=3 (accessed 18 July 2022). Reference to a dataset [dataset], 2022, (accessed 18 July 2022). ^4^ ClinicalTrials.gov. Study Record Detail. https://clinicaltrials.gov/ct2/show/NCT03305419?cond=GSK2982772&draw=2&rank=4 (accessed 18 July 2022). Reference to a dataset [dataset], 2022, (accessed 18 July 2022). ^5^ClinicalTrials.gov. Study Record Detail. https://clinicaltrials.gov/ct2/show/NCT02776033?cond=GSK2982772&draw=2&rank=5 (accessed 18 July 2022). Reference to a dataset [dataset], 2022, (accessed 18 July 2022). 6 ClinicalTrials.gov. Study Record Detail. https://clinicaltrials.gov/ct2/show/NCT03681951?cond=GSK3145095&draw=2&rank=1(accessed 18 July 2022). Reference to a dataset [dataset], 2022, (accessed 18 July 2022). ^7^ClinicalTrials.gov. Study Record Detail. https://clinicaltrials.gov/ct2/show/NCT03757325?cond=DNL747&draw=2&rank=1(accessed 18 July 2022).

#### 4.4.2 Hybrid-type RIPK1 kinase inhibitor

In 2015, Najjar et al. obtained a “hybrid” RIPK**1** inhibitor PN**10** (Figure 19B) by combining the anticancer drug ponatinib as a skeleton and covalently combining Nec-1s,. The IC_50_ value of RIPK1 *in vitro* was 108.0 nM (Najjar et al., 2015). The study on the efficacy of PN**10** *in vivo* showed that PN**10** can effectively block the injury induced by TNF-α. Compared with Nec-1s, these hybrids improved the activity of RIPK**1** and maintained higher kinase selectivity, but the higher molecular weight made these inhibitors not conducive to the optimization of lead compounds.

Ponatinib had good activity against both RIPK**1** and RIP3 kinases. The substitution of methyl on the A ring weakened the inhibition of all three RIPs and Abl due to its hydrophobic effect in the lipophilic pocket. With the introduction of larger substituents in the A ring, the selectivity to RIPK**1** kinase increased, while the inhibitory activity decreased. This is because, on the one hand, the DLG pocket of RIPK**1** was more flexible and accommodated large substituted groups connected to ponatinib’s A ring; on the other hand, Met92 Gatekeeper restricted the activities of the binding pocket to reduce the inhibitory effect on RIPK**1**. Therefore, based on the size difference of the kinase binding pocket, the selectivity and inhibitory activity of the compound to could be predicted. Compared with Abl, RIP2, and RIP3, RIPK**1** contained a smaller hydrophobic pocket and accommodated the methyl group of the A ring. Therefore, RIPK**1** and RIP2/Abl had potential differences in the binding sites around the benzene ring of ponatinib. The scaffold structure with a high affinity for RIPKs was retained and modified to enhance its selectivity to RIPK**1**. The eutectic structure of RIPK**1**/Nec-**1** showed that Nec-**1** had a “kink” conformation in DLG-out pocket, with several specific binding points in the pocket, but this contact was excluded by the narrower Glu-in conformation in the RIPK**1**/ponatinib docking model. The flat hydrophobic part in ponatinib coincided well with the narrower conformation of Glu-in/DXG-out, so the component of ponatinib connected to the conformation of Glu-in/DXG-out was retained. The low selective component of ponatinib binding to DLG pocket was replaced with the more selective component of Nec-1 (Najjar et al., 2015). The binding mode of these inhibitors to RIPK**1** had not been reported. However, it was speculated that PN**10** was unlikely to bind to the conformation of DLG-out, because this might lead to spatial conflict between the Met67 residue in RIPK**1** α-C-helix and the hydantoin of Nec-**1**.

#### 4.4.3 Benzyl-1h-pyrazoles

Zou et al. obtained RIPK**1** kinase inhibitor 38 containing 1-benzyl-1H-pyrazole through compound library screening (Zou et al., 2016). Compound **4b** was obtained by optimizing the structure of the lead compound **1a** (Figure 19C). The EC50(HT-29) value of compound **4b** in programmed necrosis inhibition test of HT-29 cells was 0.160 μM, and the Kd value of compound **4b** to RIPK**1** kinase was 0.078 μM. In the mouse model of l-arginine-induced pancreatitis, compound **4b** had a significant protective effect on the pancreas. Substituents on the benzene ring were very important for anti-necrotic activity, and the number and position of halogen atoms on the benzene ring affected the biological activity of these compounds. Substitution of a benzene ring with a benzene heterocyclic or biphenyl leads to a decrease in biological activity, with pure benzyl (without any substituents) leading to a decrease in activity. The activity of compounds containing a substituted ortho-, meta- or parachloride was also 1–3 times lower than that of 1A. When the p-chlorine atom is replaced by the more polar methoxyl, amino, or trifluoromethyl groups, the biological activity is further reduced. Compounds containing a substituted p-bromine and o-methyl also show reduced biological activity. The compound **1q** with two fluorine substituents at positions 2 and five of the benzene ring had the strongest activity [EC_50_ (HT-29) = 0.290 μM]. The introduction of chlorine or bromine atoms into the C-4 position of the pyrazole ring of compound **1q** improved the biological activity slightly, and finally compound **4b** was confirmed.

The binding mode of compound **4b** showed that compound **4b** properly occupied the allosteric pocket of RIPK**1** kinase, forming two hydrogen bonds between 4-bromo-3-nitro-1h-pyrazole part and RIPK**1** kinase residue Asp156, and 2,5-difluorobenzene ring was located in a hydrophobic bag composed of hydrophobic residues such as Leu157, Ala155, Val76, Ile154 and Met92 (Zou et al., 2016). Although these inhibitors have a relatively new skeleton, the eutectic structure of these inhibitors and RIPK**1** has not been further reported.

#### 4.4.4 GSK′963

Berger et al. obtained chiral small molecule inhibitor GSK′**963** (Figure 19D) through high-throughput screening, which effectively blocked the programmed necrosis of mouse L929 cells and human U937 cells with IC_50_ values of 1.0 nM and 4.0 nM, respectively (Berger et al., 2015). GSK′**963** was isolated from an identified racemic compound, and its enantiomeric GSK′962 (Figure 23) had no pharmacological activity. GSK′**963** effectively inhibited hypothermia in the TNF-induced shock model and avoided the effect of hypothermia on mice. Although the activity and selectivity were high, the oral exposure of these inhibitors to rodents was very low, which limited the development of these inhibitors as tool compounds. Similarly, the protein binding patterns of these inhibitors have not been reported.

## 5 RIPK1 kinase inhibitors on the market and in preclinical and clinical stages

Many RIPK**1** inhibitors are commercially available. Should be there are no clinically approved RIPK**1** inhibitors on the market so far. Currently in the clinical stage of RIPK**1** kinase inhibitors included GSK**2982772**, GSK**3145095**, and DNL**747**.

### 5.1 GSK2982772

GSK**2982772** was an effective ATP competitive RIPK**1** kinase inhibitor with oral activity, and the IC_50_ value of its inhibitory effect on human RIPK1 was 1 nM. GSK**2982772** was mainly distributed in the colon, liver, kidney and heart, and the tissue concentration was similar to that in blood, and the permeability of the GSK**2982772** cell membrane was good, but it was low in rat brain (Berger et al., 2015). Because of its good physical and chemical properties, pharmacokinetic, and high efficiency to RIPK**1**, GSK**2982772** was given orally at a low dose. At present, the **compound** is in phase II clinical study, and the indications are plaque psoriasis, rheumatoid arthritis, and ulcerative colitis. In addition, the inhibitor has completed phase I clinical studies of inflammatory bowel disease.

### 5.2 GSK3145095

GSK**3145095** was a benzoxazepine drug further optimized and developed by GlaxoSmithKline Company according to the structure of GSK**2982772**. It had good metabolic stability *in vitro* and was removed from the body by hydroxylation and glucosylation *in vivo*. It had high bioavailability, low clearance rate, and a half-life of about 3.3 h (Harris et al., 2019b). The clinical trial of GSK**3145095** was terminated following an internal review of the company’s current research and development portfolio.

### 5.3 DNL747 ↗

DNL747 is able to penetrate the blood-brain barrier. DNL747 was discontinued in Phase I in July 2020 due to long toxicity. In addition to DNL47, another RIPK**1** inhibitor, DNL758, is undergoing early clinical evaluation (Dannappel et al., 2014). DNL758 has no blood-brain barrier penetration and is used in Cutaneous Lupus Erythematosus.

## 6 Conclusion and prospect

Most previous studies has shown that RIPK**1** kinase activity can gather a variety of signal inputs, induce regulated cell death, and thus mediate tissue injury and inflammation (Linkermann and Green, 2014). Besides RIPK**1**, RIP3 was also an essential factor leading to programmed necrosis (Vanlangenakker et al., 2012; Newton et al., 2014). RIP3 activated the necrotic pathway in the absence of RIPK**1**, this suggested that RIP3 was essential for programmed necrosis, whereas RIPK**1** was not. The new study found that RIP3 oligomerization was sufficient to induce necrosis in the absence of TNF stimulation and RIPK**1** activity (Orozco et al., 2014). Therefore, RIP3 was the key to regulate necrosis. As mentioned above, the interaction between RIPK**1** and RIP3 led to autophosphorylation and dephosphorylation within RIPK**1**/RIP3. Then phosphorylated RIP3 was recruited and phosphorylated MLKL. However, the dominant function of RIPK**1** did not always promote RIP3, and it was also involved in cell protection (Liu et al., 2019). Jaco et al. reported MK2 phosphorylated RIPK**1** at Ser321 to block TNF-induced cell death (Jaco et al., 2017). In recent years, a variety of molecules and mechanisms related to programmed necrosis has been found. In addition, since the function of RIPK1 cells is regulated by kinases such as TAK1, IKK, MK2 and TBK1, drugs affecting these kinases may also lead to changes in ripK1-mediated necrosis pathways. Studies have found that RIPK**1** directly receives oxidative regulation by ROS, thus enhancing kinase activity and resulting in autophosphorylation of Ser161 (S161). ROS production depends on the function of RIPK**3** in the necrosome, so ROS mediates positive feedback regulation in the programmed necrosis pathway (Zhang et al., 2017). Due to the lack of in-depth biological research, the regulatory mechanism of RIPK**1**-related programmed necrosis signal pathway in various diseases and the changes of downstream regulatory molecules of RIPK**1** need to be further studied. In addition to programmed necrosis, RIPK**1** was also involved in TNF-induced apoptosis when NF-κB signal pathway was inhibited. Caspase8 inhibited the activation of RIPK**1**. But it is not clear how RIPK**1** kinase activity regulates the activation of caspase (Zarrin et al., 2021).

Nec-1 is a commonly used kinase inhibitor tool in the field of necrosis research, but the medium potency of Nec-**1** and its targeting activity to IDO make it not the best kinase inhibitor tool. The immune tolerance pathway is activated in cancer tissues, and IDO activity is related to tumor immune tolerance. Nec-1 targeting IDO has mixed activity. Nec-1s is a relatively potent and specific inhibitor of RIPK1 kinase that was used in the study. GNE684 has a strong effect on human RIPK1 with high specificity and is a good research tool. The multi-targeted kinase inhibitor Ponatinib, as an antitumor agent, can also prevent necrotic ptosis by targeting RIPK**1**, but its lack of specificity and severe long-term adverse reactions have limited its development for the treatment of inflammatory diseases associated with RIPK1. In addition, in the study of kinase inhibitors, real-time activation or necrosis markers of RIPK1 need to be tracked *in vivo*. Therefore, the selectivity of RIPK**1** kinase inhibitions must be considered in the study of RIPK**1** kinase inhibitions, especially the selectivity of AURK kinase and other kinases in RIPK family. The ideal kinase inhibitor needs to have strong selectivity and minimal off-target kinase activity. Type Ⅲ kinase inhibitions have no hinge binding interaction and occupy an allosteric lipophilic pocket on the back of the ATP binding site. Therefore, type Ⅲ kinase inhibitions are ideal. The research of RIPK**1** kinase inhibitions has made important progress, but there are still many problems to be solved, such as poor pharmacokinetic properties, metabolic instability, and low oral bioavailability. Further structural modifications can be made on the basis of existing compounds to improve their pharmacokinetic properties and make them more suitable for clinical use. The development of RIPK**1** inhibitions with better activity, selectivity, and pharmacokinetic properties is the focus of future research. With the in-depth development of programmed necrosis pathway research, it is expected that more and more new RIPK**1** kinase inhibitors with good biological activity and small side effects will be found in the programmed necrosis pathway as therapeutic drugs.

## Glossary

TNF: Tumor necrosis factor
TRAILR: TNF related apoptosis inducing ligand receptor
TNFR: TNF receptor
FADD: Fas-associated death domain
TRADD: TNF receptor-associated death domain
RHlM: RIP homotypic interaction motif
TRAF2: TNF receptor-associated Factor 2
cIAP1: cellular inhibitors of apoptosis 1
cIAP2: cellular inhibitors of apoptosis 2
LUBAC: linear ubiquitin assembly complex
IKK: Ikappa B kinase
TAK: TGFβ-activated kinase
TAB: TAK1, and TAK1 binding protein
NF-κB: nuclear factor-kappa B
CYLD: Cylindromatosis
NEMO: NFκB essential modulator
MLKL: mixed lineage kinase domain-like protein
NoX-4: Nicotinamide Adenine Dinucleotide Phosphate Oxidase 4
IL-6: Interleukin-6
IL-8: Interleukin-8
HMGB1: High Mobility Group Protein B1
IL-1β: Interleukin-1β
DRP1: dynamic related protein 1: dynamic related protein 1
BMDMs: bone marrow derived macrophages
DRP1: dynamic related protein 1: dynamic related protein 1
MCAO/R: middle cerebral artery occlusion/reperfusion
MAP2: microtubule-associated protein 2
GFAP: glial fibrillary acidic protein
I/R: ischemia/reperfusion
APP: amyloid precursor protein
PS1: premature aging protein 1
DAM: disease-associated microglia
AD: Alzheimer’s disease
AurK: Aurora kinase
SIRS: Systemic Inflammatory Response Syndrome.
