# Erratum: Advances in RIPK1 kinase inhibitors

**DOI:** 10.3389/fphar.2022.1123234

**Published:** 2023-01-09

**Authors:** Frontiers Production Office

**Affiliations:** Frontiers Media SA, Lausanne, Switzerland

**Keywords:** inhibitor, receptor interacting protein 1 (RIP1), programmed necrosis, necrosis, RIP1 (RIPK1)

Due to a production error, the list of affiliations used for the authors was incorrect. The correct affiliations list is shown below.

Lu Chen^1,2,†^, Xiaoqin Zhang^3,†^, Yaqing Ou^4,†^, Maoyu Liu^1,2,†^, Dongke Yu^1,2^, Zhiheng Song^5^, Lihong Niu^6,^*, Lijuan Zhang^1,2,^* and Jianyou Shi^1,2,^*

^1^Department of Pharmacy, Sichuan Academy of Medical Sciences & Sichuan Provincial People’s Hospital, School of Medicine, University of Electronic Science and Technology of China, Chengdu, China

^2^Personalized Drug Therapy Key Laboratory of Sichuan Province, School of Medicine, University of Electronic Science and Technology of China, Chengdu, China

^3^Department of Critical Care Medicine, Sichuan Academy of Medical Sciences & Sichuan Provincial People’s Hospital, Affiliated Hospital of University of Electronic Science and Technology of China, Chengdu, Sichuan, China

^4^Department of Pharmacy, The Affiliated Chengdu 363 Hospital of Southwest Medical University, Chengdu, Sichuan, China

^5^Suzhou University of Science and Technology, Suzhou, Jiangsu, China, 6 Institute of Laboratory Animal Sciences, Academy of Medical Sciences and Sichuan Provincial People’s Hospital, Chengdu, Sichuan, China

The publisher apologizes for this mistake. The original version of this article has been updated.

